# Origin and evolution of colorectal mixed neuroendocrine–non-neuroendocrine neoplasms (MiNEN)

**DOI:** 10.1530/ERC-26-0170

**Published:** 2026-07-27

**Authors:** Siren Morken, Halfdan Sorbye, Wei Deng, Hatice Toprak Dogramaci, Andreas Venizelos, Lene Weber Vestermark, Per Pfeiffer, Christian Kersten, Aurel Perren, Stian Knappskog

**Affiliations:** ^1^ Cancer Clinic, Haukeland University Hospital, Bergen, Norway; ^2^ Department of Clinical Science, University of Bergen, Bergen, Norway; ^3^ Department of Oncology, University Hospital of Southern Denmark, Esbjerg, Denmark; ^4^ Department of Oncology, Odense University Hospital, Odense, Denmark; ^5^ Department of Research, Hospital of Southern Norway, Kristiansand, Norway; ^6^ Institute of Tissue Medicine and Pathology, University of Bern, Bern, Switzerland

**Keywords:** colorectal, MiNEN, NEC, origin, mutation

## Abstract

**Graphical Abstract:**

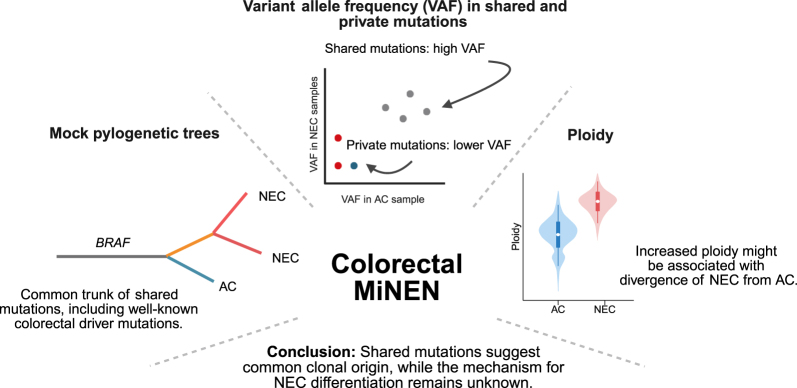

**Abstract:**

Colorectal neuroendocrine carcinoma (NEC) is a rare and aggressive cancer and in a subset of patients associated with an adenocarcinoma (AC) component. When both components exceed 30% of the tumour, it is classified as mixed neuroendocrine–non-neuroendocrine neoplasm (MiNEN), although there is an ongoing debate about whether any presence of two distinct components should be sufficient for a MiNEN diagnosis. This study aimed to investigate the origin and subsequent genetic changes of these two components. Ten colorectal cases suitable for sampling of an AC and a poorly differentiated NEC component were identified from the NORDIC NEC 2 study and sequenced across a 360-cancer gene panel. Mock phylogenetic trees were constructed from the molecular profiles of each sample within a patient. All ten cases revealed a common trunk of shared somatic mutations, including well-known colorectal cancer driver mutations such as *BRAF*, *KRAS*, *APC*, and *TP53*. In all cases, a single branching point separated the AC and NEC components. Private AC and NEC mutations generally had low variant allele frequencies, indicating that most AC and NEC cells were genetically similar. NEC, when compared with AC samples, demonstrated a higher frequency of private mutations (*P* = 0.009), indicating a higher mutation rate and greater ploidy (*P* = 0.012), suggesting an association between genomic duplication and AC-to-NEC transition. Shared mutations indicate a common clonal origin, underscoring the role of established colorectal driver mutations in the early development of these tumours, while the mechanisms underlying NEC differentiation remain poorly understood and may involve non-genetic factors.

## Introduction

Colorectal neuroendocrine carcinoma (CR-NEC) is a rare and aggressive disease and in a subset of cases associated with a non-neuroendocrine component. Digestive tumours composed of a neuroendocrine and a non-neuroendocrine component are formally classified as mixed neuroendocrine–non-neuroendocrine neoplasm (MiNEN) if both components exceed 30% of the lesion (1). However, there is an ongoing debate about whether any presence, regardless of size, of two distinct components is sufficient to define the tumour as MiNEN (2). Digestive MiNEN are most commonly found in the colorectum in Western populations and usually consist of a NEC and adenocarcinoma component. Previously, these tumours were referred to as mixed adenoneuroendocrine carcinoma (MANEC) (3, 4, 5).

While the classification of digestive NEC and MiNEN is formally established, there are significant grey areas in the classification of these tumours (2), and the underlying biology of MiNEN remains poorly understood. A better understanding of the genetic changes during MiNEN evolution may inform future treatment strategies, which are currently controversial in digestive MiNEN (6). In recent years, the molecular landscape of digestive NEC has been more elucidated, highlighting the molecular similarities between CR-NEC and colorectal adenocarcinoma (CR-AC) (7, 8, 9, 10). These molecular similarities and their frequent coexistence raise the question of whether CR-NEC and CR-AC have a shared mechanism for tumourigenesis, and in the case of CR-MiNEN, a shared clonal origin. Various theories have been proposed to explain the origin and genetic evolution of the two components in CR-MiNEN. These theories suggest that they either emerge entirely independently and combine into a single tumour or that they share a common clonal origin (3, 11). The pioneering study by Vortmeyer et al. identified identical allelic loss of key colorectal driver genes in the NEC and AC components of poorly differentiated CR-NEC, suggesting that these components arise from the same cell of origin (12). Three more recent studies exploring shared mutations in CR-NEC with an adenocarcinoma component provide support for the hypothesis of a common clonal origin of the two components (13, 14, 15). However, the potential role of genetic alterations as the underlying causes of the development of digestive NEC from a common precursor is unknown. Given a common clonal origin, multiple mechanisms for phenotypic divergence of the two components are possible, including evolvement through bi-phenotypic differentiation from a common endoderm-derived epithelial precursor cell or the NEC component evolving through dedifferentiation from a fully developed AC.

If there are CR-MiNEN cases in which the NEC and AC components develop independently and merge into a single tumour, one would not expect notable overlap in their genetic alterations. However, if the two components have a common clonal origin, there should be evidence of shared somatic mutations. In this study, we performed multi-region sequencing using a 360-cancer gene panel on both a NEC and an AC component of variable proportion, each suitable for sampling, from tumours with a colorectal primary site. In each patient, the mutational profiles of the components were compared to identify shared and private mutations, thereby assessing the potential common origin and the extent of divergence.

## Materials and methods

### Patients and samples

The set of patient samples for this study was obtained from the prospective NORDIC NEC 2 study, which included patients diagnosed with digestive high-grade neuroendocrine neoplasm (HG-NEN) during 2013–2017. The last follow-up was in 2021. Inclusion in the study required a histologically confirmed diagnosis of either pure NEC (>70% NEC component) or MANEC (>30% NEC and >30% AC component), all with a Ki-67 > 20%.

For this work, we identified 12 colorectal cases with documented morphology showing both an AC and a poorly differentiated NEC component, for which both components were available for tumour tissue sampling. No cases of well-differentiated NET G3 were included in this study. Each case was re-evaluated pathologically according to the 2019 World Health Organization classification after their inclusion in the registry (2021–2022), by experienced NEN pathologists, without access to clinical information. Out of these, eight cases were classified as MiNEN, while four cases were classified as large-cell NEC due to having an AC component below 30%, which did not meet the formal criteria for a MANEC/MiNEN diagnosis. All 12 cases were included in this study, regardless of their diagnosis, on the basis of having adequate material available for sampling from both the AC and NEC components. For the purpose of this study, all cases are hereafter referred to as CR-MiNEN. Following the sampling, a study-specific evaluation was conducted on cases with available histology reports and/or tumour tissue to confirm that samples were taken from AC and NEC components.

**Figure 1:**
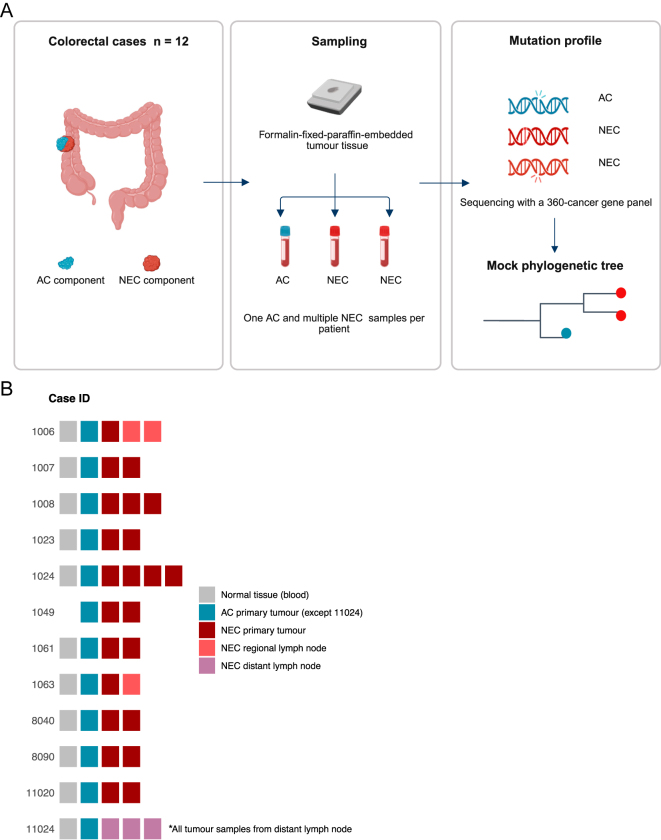
(A) Study workflow of 12 cases with both adenocarcinoma (AC) and neuroendocrine carcinoma (NEC) components, suitable for sampling, from tumours with a colorectal primary site. Samples were collected from formalin-fixed-paraffin-embedded (FFPE) tumour tissue blocks. Mutations were called from massive parallel sequencing of a 360-cancer gene panel and used to determine mock phylogenies. (B) Overview of analysed samples for all 12 cases. For all cases (except 1049), a blood sample (grey) was used as a reference. For all cases, one sample from the primary adenocarcinoma component (blue) was analysed (except for case 11024, where samples were taken from a distant lymph node). For all cases, two to four samples were taken from NEC components (primary tumour in dark red, regional lymph node in light red, or distant lymph node in purple).

The workflow for the study is illustrated in Fig. 1A. Tumour slides, formalin-fixed-paraffin-embedded (FFPE) tumour tissue, and matched blood DNA were collected for all cases. For each patient, tissue cores of 1 mm diameter × 2–3 mm depth were collected. Sampling was performed from one area with AC morphology and multiple areas with NEC morphology (Supplementary Fig. 1 (see section on Supplementary materials given at the end of the article)). When available, sampling was performed from the primary tumour (Fig. 1B). In two cases, where multiregional sampling of the NEC primary tumour was not feasible, sampling was performed from regional lymph nodes. Case 11024 is based solely on AC and NEC tumour tissue from a distant lymph node. All AC samples, except for case 11024, were collected from the primary tumour.

### Molecular analysis

DNA isolation and sequencing procedures have been reported previously (16), and details are given in Supplementary Data. In brief, DNA was isolated using ultrasonication and column-based clean-up. Mutation analyses were performed by targeted sequencing of a 360-cancer gene panel (17), captured using a custom-made Agilent SureSelect XT-kit and run in-house on the Illumina MiSeq platform. In this analysis, the entire coding regions of all genes were covered. Mapping and mutation calling were performed on the Dragen Bio-IT platform v.3.9.5. Copy number alteration (CNA) analysis and estimation of tumour cell purity and ploidy were performed using FACETS (18). MSI status was obtained through the Promega MSI analysis system (version 1.2, Promega, USA), as previously described (16) (*n* = 7), and from local routine diagnostics (*n* = 5). Further details on molecular methods, data processing, mutation calling, and calling of colorectal driver mutations (in *BRAF, KRAS, APC* and *TP53*) are given in Supplementary Data and Supplementary Table 1.

In the present sample set, molecular data from a single NEC tissue sample and matched blood DNA have been previously reported for three cases (1006, 1008, and 1023) (16, 19, 20).

### Construction of mock phylogenetic trees

To assess the clonal origin between AC and NEC samples from the same patients, we constructed mock phylogenetic trees for each patient using the R package ggtree. In this set-up, the common trunk of the trees illustrates mutations shared among all the AC and NEC samples from an individual patient. The outer branches illustrate private mutations found in only in the AC or one of the NEC samples in the individual patient. Branching with mutation annotation was performed solely on the presence or absence of mutations in sampled areas, without consideration of variant allele frequency (VAF). As such, the trees represent geographical differences rather than necessarily subclonal evolution (hence the term ‘mock phylogenetic tree’). We performed manual post hoc mutation calling for trees with non-logical initial solutions (Supplementary Data). Here, we performed manual inspection of sequencing reads to search for mutations with VAF below the threshold required for the original formal mutation calling. In cases where such mutations were found, these manual calls were retrieved to construct the trees (marked by grey font in the illustrations).

### Comparison of molecular features

We compared the frequency of private mutations in patients with available AC and NEC primary tumour samples. In cases with multiple NEC samples, the mean number of private NEC mutations was calculated and the number of NEC-specific truncal mutations was added to create a single NEC value for comparison with the corresponding AC sample. Ploidy-adjusted copy numbers (copy number minus ploidy) were assessed across the gene panel in each sample. Identical CNAs across an AC and a NEC sample within a patient were defined as truncal CNAs. CNAs that were altered in only the AC or NEC sample, or if the CNA was numerically different between the AC and NEC sample, were defined as branch CNAs (see Supplementary Data for details). In addition, we compared the level of non-ploidy-adjusted copy numbers for selected genes in the AC and NEC components within each patient. We also assessed ploidy across AC and NEC primary tumour samples, comparing the mean ploidy of the NEC samples with that of the corresponding AC sample.

### Statistics

Descriptive statistics were used for baseline characteristics. Paired samples of continuous variables were compared using the Wilcoxon signed-rank test. Statistical analyses and figures were created using R, version 4.5.2.

### Ethics

The NORDIC NEC 2 study was approved by regional ethics committees in Denmark, Sweden, and Norway. The study was conducted in accordance with the Declaration of Helsinki, and written informed consent was obtained from all patients.

## Results

### Patients

Twelve cases with both an AC and a poorly differentiated NEC component suitable for sampling were initially identified for this study, including eight cases of colorectal MiNEN and four cases of large-cell NEC. However, a study-specific evaluation of the sampled areas could not confirm the NEC component in two cases (1024 and 1049). Instead, these re-evaluations suggested a mixed morphology of classic AC and AC with synaptophysin expression (AC with neuroendocrine differentiation) (21). Consequently, these two cases were omitted from the main analysis. In patient 1008, the study-specific evaluation also favoured adenocarcinoma histology, with synaptophysin expression in one of three NEC samples (NEC-C). Despite acknowledged uncertainty, the NEC label was retained for this sample based on the initial assessment and the expression of both synaptophysin and chromogranin A, leaving a main study cohort of ten cases. The clinicopathological characteristics of all 12 included cases are shown in Table 1.

**Table 1:** Clinicopathological characteristics of 12 colorectal cases with a described neuroendocrine carcinoma (NEC) and adenocarcinoma (AC) component suitable for tumour tissue sampling.

Patient	Sex	Age (years)	Primary site	Diagnosis (Ki-67)	Study-specific verification of NEC and AC component	Metastatic disease	Metastatic sites	OS from diagnosis (months)	Treatment and response	Status at last follow-up	MSI status
1006	F	90	Colon left	NEC LC (70%)	Yes	No	-	105.4	No (old age)	Dead (other causes)	MSS
1007	F	68	Colon left	MiNEN (90%)	Yes	Metachronous	Liver	14.4	Adjuvant: EP 1-line: TEMCAP (PD)	Dead (NEC)	MSS
1008	M	71	Colon right	NEC LC (70%)	Yes	Synchronous	Liver	10.3	1-Line: EP (PD) 2-line: CAPTEM (PD) 3-line: FLIR (SD) 4-line: FLOX (PD)	Dead (NEC)	MSS
1023	M	75	Rectum	NEC LC (50%)	Yes	Metachronous	Liver, lung, bone	21.8	Adjuvant: EP 1-line: EP (NA)	Dead (NEC)	MSS
1024	F	75	Colon right	MiNEN (70%)	No: AC with synaptophysin expression	Synchronous	Lymph nodes, lung	61.4	No (patient request)	Dead (other causes)	MSI-H
1049	F	82	Colon right	NEC LC (80%)	No: AC with synaptophysin expression	Metachronous	Liver	10.2	Adjuvant: FLOX	Dead (NEC)	MSS
1061	M	50	Rectum	MiNEN (70%)	Yes	Metachronous	Liver	76.7	Secondary liver resection (with neoadjuvant/adjuvant: EP)	Alive	MSS
1063	M	74	Colon right	MiNEN (50%)	Yes	Metachronous	Liver, lung	5.8	No (poor PS)	Dead (NEC)	MSS
8040	F	73	Colon right	MiNEN (80%)	Yes	Metachronous	Liver, lung	7.3	No (poor PS)	Dead (NEC)	MSS
8090	F	61	Rectum	MiNEN (80%)	Yes	Metachronous	Liver, lung, mammae	17.0	Neoadjuvant/adjuvant: EP 1-line: EP (PD) 2-line: CAPTEM (SD)	Dead (NEC)	MSS
11020	M	76	Colon right	MiNEN (80%)	Yes	No	-	82.3	Adjuvant: EP	Alive	MSI-H
11024*	F	76	Colon right (adenocarcinoma)	MiNEN (80%)	Yes	Metachronous	Lymph nodes, liver, lung, brain, skin	2.4	No (poor PS)	Dead (NEC)	MSS

EP, platinum/etoposide; F, female; FLIR, fluorouracil/irinotecan; FLOX, fluorouracil/oxaliplatin; LC, large cell; M, male; MiNEN, mixed neuroendocrine–non-neuroendocrine neoplasm; MSI, microsatellite instability; MSI-H, microsatellite instability-high; MSS, microsatellite stable NA, not assessed; NEC, neuroendocrine carcinoma; OS, overall survival; PD, progressive disease; PS, performance status; SD, stable disease; TEMCAP, temozolomide/capecitabine.

* Diagnosed and resected for adenocarcinoma in the colon two and a half years prior to the metastatic MiNEN diagnosis. OS is calculated from the time of MiNEN diagnosis.

### The evolutionary pattern

To assess the origin and evolutionary relationship between the two components in CR-MiNEN we performed next-generation sequencing with a 360-cancer gene panel of one AC sample and two or three NEC samples (depending on the size of the area suitable for sampling; Fig. 1B). Shared and private mutations for samples from individual patients were used to construct mock phylogenetic trees.

**Figure 2:**
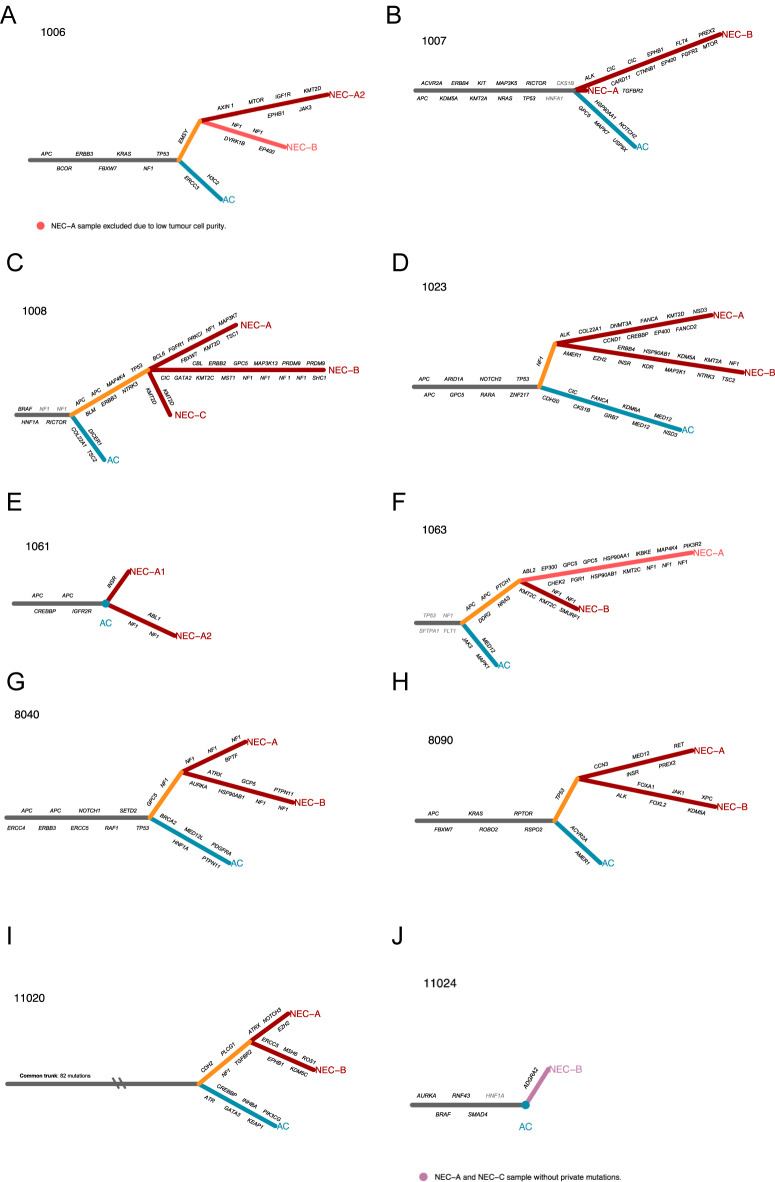
Mock phylogenetic trees. Trees based on multiregion analyses of ten cases with colorectal mixed neuroendocrine–non-neuroendocrine neoplasm (MiNEN). Mutations are annotated to their respective branches. Mutations with variant allele frequency below the threshold required for the original formal mutation calling are marked in grey font. Grey lines indicate a common trunk with mutations detected in all samples. Blue lines indicate mutations private to adenocarcinoma (AC). Orange lines indicate mutations on a neuroendocrine carcinoma (NEC)-specific trunk, while dark and light red lines indicate mutations private to NEC primary tumour and NEC in lymph nodes, respectively. Purple line indicates private mutations to NEC in distant lymph nodes.

All ten cases displayed a tree pattern with a common trunk of shared mutations, i.e. mutations present in all AC and NEC samples from an individual patient (Fig. 2A, B, C, D, E, F, G, H, I, J). A very clear pattern was seen while assessing the branch structure of the trees. In all cases, the common trunk of shared mutations was followed by a single point of split between the AC and NEC samples. Among these, seven cases had a sub-pattern in which the split between the AC and NEC branches was followed by further sub-branching of the individual NEC samples (e.g. patients 1006 and 1008). In one case (patient 1007), there was simultaneous branching of AC and NEC from the common trunk, and in two cases (patients 1061 and 11024), private mutations were detected in the NEC samples but none in the AC samples.

The two cases displaying a synaptophysin-expressing adenocarcinoma, rather than a NEC component, exhibited similar tree structures, with a common trunk of shared mutations, followed by a single split into branching of private mutations in the classic AC and synaptophysin-expressing AC component.

### Time of split and mutation rates

**Figure 3:**
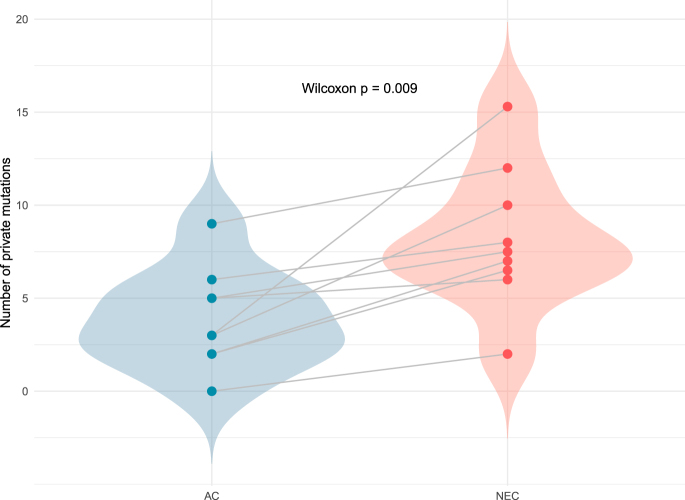
Private mutations. Matched (intra-patient) comparison of the number of private mutations in the adenocarcinoma (AC) versus the neuroendocrine carcinoma (NEC) (mean across samples per patient; see the section titled ‘Methods’) component in nine cases with available primary tumour samples. Since all tumours revealed a single time of split between the AC and NEC components and the samples were collected at the same time, differences in the number of mutations indicate differences in mutational rate.

Based on the proportion of shared versus private mutations, the molecular time for the split between AC and NEC varied largely between patients. For example, patient 1063 had a tumour with few shared mutations and a relatively large proportion of private mutations, indicating an early split, while, e.g. 11020 had an extremely large trunk and relatively few private mutations, indicating a late split. Of note, patient 11020 was the only MSI-H case in the cohort. Looking at the mutational evolution after the time of split in primary tumour samples, there was a significant higher proportion of private NEC mutations than private AC mutations (*P* = 0.009, Fig. 3). Given a single time of split and sampling at the same time point, this shows that the mutation rate is higher in the NEC regions than in the AC region.

**Figure 4:**
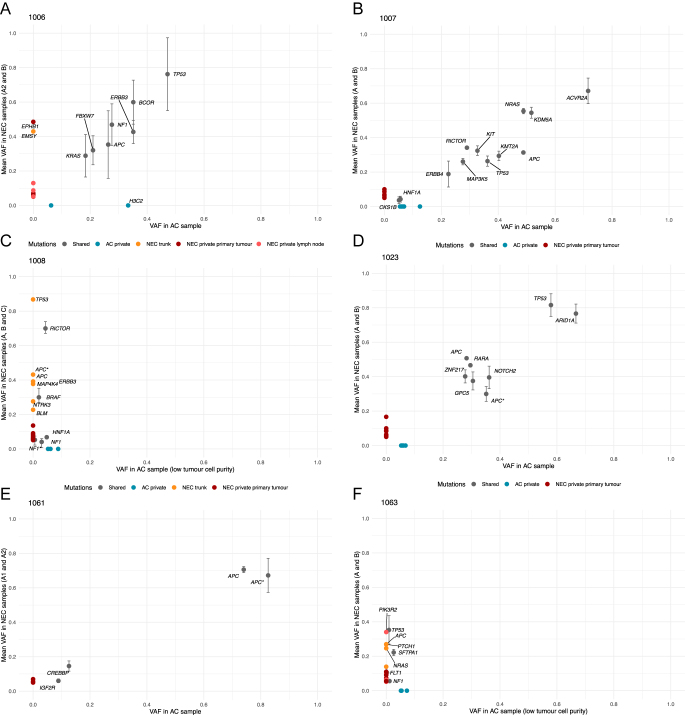

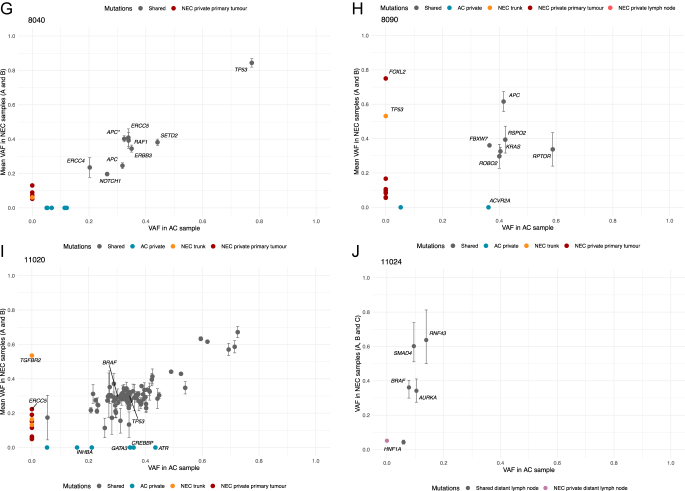
Variant allele frequencies of mutations. Scatterplots illustrate all detected mutations in ten cases with colorectal mixed neuroendocrine–non-neuroendocrine neoplasm. Mutations are plotted as dots according to their variant allele frequency (VAF) in the adenocarcinoma (AC) sample (x-axis) and the VAF in NEC samples (y-axis). For VAF in the neuroendocrine carcinoma (NEC) samples, the dots represent the mean VAF, and the whiskers represent the range of VAFs for all NEC samples in the respective patient. Grey dots indicate mutations shared across all samples in a patient. Blue dots indicate mutations private to AC. Orange dots indicate mutations on a NEC-specific trunk, while dark and light red dots indicate mutations private to NEC primary tumour and NEC regional lymph nodes, respectively. Purple dots indicate private mutations to NEC in distant lymph nodes.

All cases exhibited shared canonical colorectal driver mutations (*BRAF* (3/10), *KRAS* (2/10), *APC* (6/10), or *TP53* (6/10)) (Supplementary Fig. 2), while *RB1* mutations were not found in any cases. In some cases, canonical driver mutations were also found in the common NEC branch at VAF indicating them to be fully clonal and, therefore, to be a driver of a full clonal sweep in the NEC component during or after the evolutionary split from the AC component. This is exemplified by the *APC* and *TP53* mutations in patient 1008 (Figs 2C and 4C) and by the *TP53* mutation in patient 8090 (Figs 2H and 4H). However, this was not common, and, in most cases, the private mutations had low VAF, indicating that only few cells in the sampled region harboured them (Fig. 4A, B, C, D, E, F, G, H, I, J). This observation, together with the generally higher VAFs of shared mutations, does not support that the private mutations are the critical molecular events causing the phenotypic split between AC and NEC. Rather, the data indicate that, in most cases, there is a high fraction of AC and NEC cells harbouring *only* the truncal mutations and that the differentiation between AC and NEC, therefore, is not related to mutations covered by the 360-cancer gene panel.

Notably, in seven of the ten cases, there was a shared NEC-specific trunk after the split from AC followed by further sub-branching of NEC, and in five of these seven, the NEC-specific trunk displayed mutations with a VAF ≥ 0.2. Due to the limited gene panel size, our data were not formally corrected for tumour cell purity or local copy numbers, but with few exceptions, the high VAF of shared mutations indicates that they were fully clonal. In several cases, shared mutations had VAF well above 0.5, indicating loss of the wild-type allele (loss of heterozygosity).

### Parallel evolution

We found several cases to harbour different mutations in the same genes in the same samples (e.g. *APC* in 8040 and *NF1* in 1061; Fig. 2), and several cases with different mutations in the same genes across different samples (e.g. *NF1* in 1008 and 8040), indicating parallel evolution. There were notable differences between the affected genes: *APC* mutations occurred early and in many cases multiple *APC* mutations were found on the trunk of the trees (1023, 1061, and 8040). *NF1* displayed parallel evolution in six cases, restricted to the NEC components and almost exclusively with low VAFs. Only case 11020 had a private *NF1* mutation with high VAF. The presence of multiple low VAF *NF1* mutations indicates that these mutations are not involved in the development of NEC, but that there is a selection pressure favouring cells with *NF1* defects in established NEC after the divergence from AC.

### Copy number changes and ploidy

**Figure 5:**
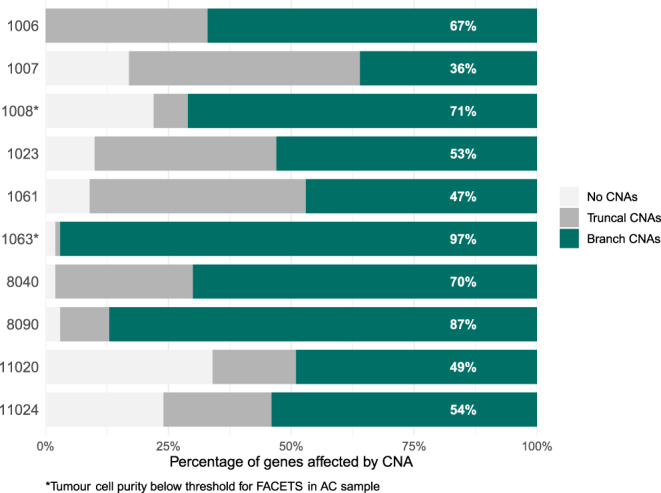
Copy number alterations. Bars illustrating the percentage of genes (within the analysed 360-cancer gene panel) affected by ploidy-adjusted copy number alterations (CNAs) occurring before the split of the neuroendocrine carcinoma (NEC) and adenocarcinoma (AC) component (truncal CNAs; grey bars) and after the split (branch CNAs; green bars) in ten cases with colorectal mixed neuroendocrine–non-neuroendocrine neoplasm.

**Figure 6:**
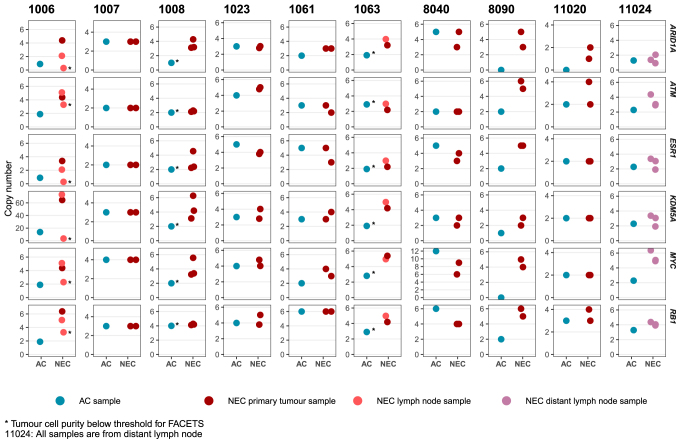
Copy number alterations in selected genes. Comparison of non-ploidy-adjusted copy number alterations in selected genes in the adenocarcinoma (AC) and neuroendocrine carcinoma (NEC) components, in ten cases with colorectal mixed neuroendocrine–non-neuroendocrine neoplasm. Blue dots indicate mutations private to AC. Dark and light red dots indicate mutations specific to NEC primary tumour and in NEC regional lymph nodes, respectively. Purple dots indicate private mutations to NEC in distant lymph nodes. The asterisk indicates samples with low tumour cell purity.

**Figure 7:**
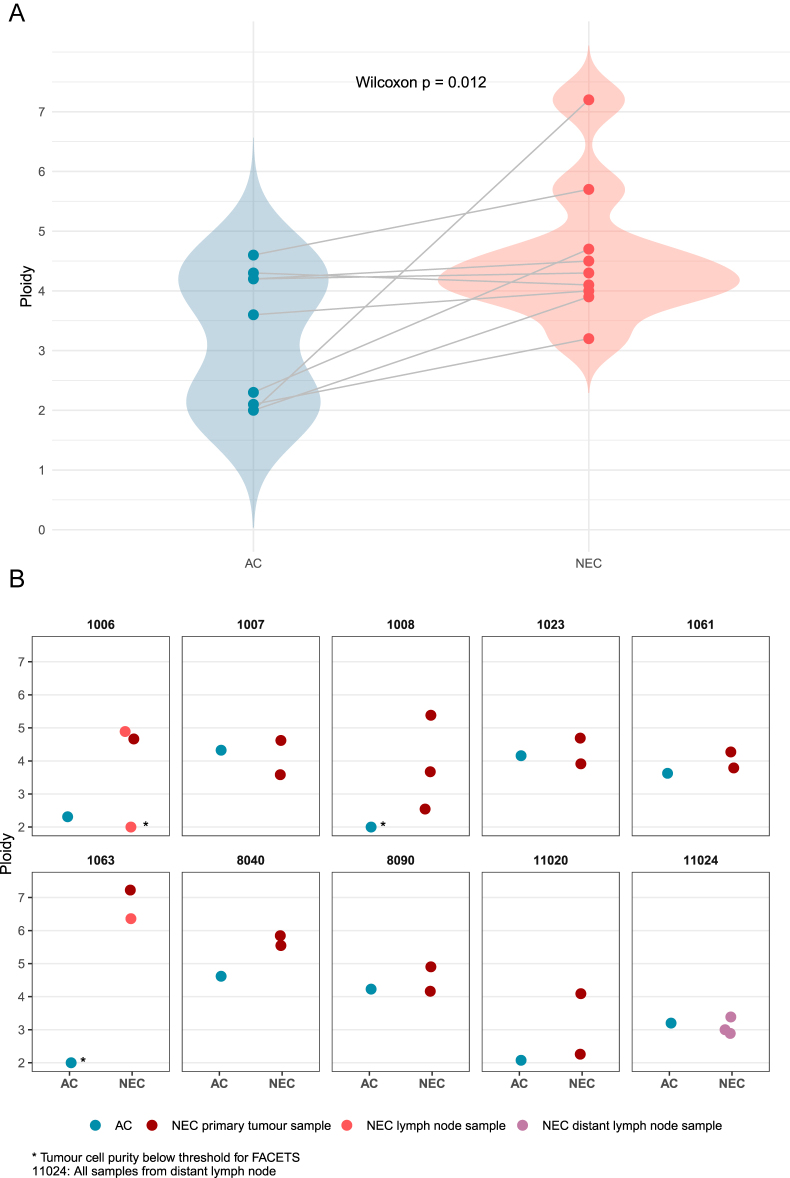
Ploidy. (A) Matched (intra-patient) comparison of ploidy in the adenocarcinoma (AC) versus the neuroendocrine carcinoma (NEC) (mean across samples per patient; see the section titled ‘Methods’) component in nine cases with available primary tumour samples. (B) Ploidy of individual samples within the ten cases with colorectal mixed neuroendocrine–non-neuroendocrine neoplasm.

We examined copy number patterns across the gene panel in each patient and found a high number of both truncal and branch CNAs, i.e. alterations occurring before and after the split of the AC and NEC components, respectively. However, there was a general enrichment of CNAs after the split, with seven out of the ten cases revealing >50% of CNAs as branch events (after split; Fig. 5). Comparing the general distribution of branch CNAs between the AC and NEC components, the highest number of CNAs was seen in AC in some cases and in NEC in other cases, without revealing any clear pattern (Supplementary Table 2). In addition, we assessed CNAs in genes that are previously reported to be the most frequently altered (*MYC*, *KDM5A*, *RB1*, *ARID1A*, *ATM*, and *ESR1*) (16). CNAs were seen in all the selected genes, and present in both the AC and NEC samples (Fig. 6). While the number and level of CNAs for these genes were in general higher in NEC than AC components, no systematic differences for any of the investigated genes, individually, were found. Of note, no *RB1* loss was detected in any cases. This indicates that the CNAs in these selected genes cannot explain the split between the AC and NEC components. On the other hand, when assessing ploidy in primary tumour samples, this was significantly higher in the NEC than in the AC component (*P* = 0.012, Fig. 7A), with the difference remaining significant even when excluding cases with low tumour cell purity (*P* = 0.047, Supplementary Fig. 3). Notably, with one possible exception (in case 1007), all NEC samples had similar or higher ploidy than their matched AC samples (Fig. 7B). As such, increased ploidy might be associated with the phenotypical divergence of NEC from AC.

## Discussion

Colorectal MiNEN is a rare and aggressive disease, with a huge lack of clinical and molecular data (3). The best treatment for patients with advanced disease remains uncertain (6). From other cancers, it is known that the tumour’s origin is one of the best predictors for treatment efficacy (22), and a better understanding of the genetic changes occurring during the evolution of CR-MiNEN might help guide future treatment of this disease. In this study, we examined the molecular profile of ten patients with CR-MiNEN. We constructed mock phylogenetic trees based on shared and private mutations to illustrate the origin and development of the two tumour components. Our data strongly support that the AC and NEC components in these tumours share a clonal origin from a common epithelial precursor cell.

All ten cases showed a tree pattern with a common trunk of shared mutations between AC and NEC components, indicating a monoclonal origin of the two components. The common trunk was followed by a single split and progression into two distinct entities with private mutations. Shared mutations included well-established alterations in colorectal tumourigenesis (*BRAF*, *KRAS*, *APC,* and *TP53*) and, in general, had higher VAFs than the private mutations, indicating that they play an important role in the early development of these tumours. Our finding of shared origin is consistent with previous studies on CR-MiNEN, which have reported molecular similarities between the two components. In 1997, Vortmeyer et al. found the same LOH in key colorectal driver genes in both the NEC and AC component of CR-NEC (12), followed by more recent studies, identifying genetic similarities between the two components in CR-NEC cases, further strengthening the theory (13, 14, 15, 23). Similar findings have been reported for gastric-NEC with an AC component (24, 25). Shared genome-wide LOH between the NEC and AC components has been reported in mixed gastric NEC (26), and genetic similarities between the NEC and AC components have recently been reported in 33 mixed gastric carcinomas (27). In a large retrospective study of digestive MANEC, an equal association between loss of MMR proteins and expression of Rb and p53 was observed in both NEC and non-NEC components (28). Taken together, these findings suggest a common origin of the two components in CR-MiNEN, as well as for gastric-MiNEN.

In our study, all ten cases displayed a tree with a sequential divergence pattern, with a clear and single split between AC and NEC, with no evidence of further NEC branches splitting from AC and with no reversal of NEC phenotype (i.e. splitting of AC branches from established NEC). Some trees had rather long trunks, while others had short ones, indicating that the molecular time from malignant transformation to split between AC and NEC can vary. Notably, we used the conventional strategy of white blood cells as matched normal tissue (not normal colorectal tissue). Therefore, our data does not allow placement of malignant transformation on the phylogenetic trees. Several canonical driver mutations might have occurred and existed in normal tissue over time before malignant transformation and it is also possible that in some cases even the split between clones expanding into AC and NEC could have occurred before malignant transformation. However, we believe the most likely scenario is that few driver mutations have occurred before malignant transformation; this transformation, in turn, has given rise to an AC, with a subsequent phenotypical divergence to NEC. Notably, Qiu *et al*. identified two distinct divergence patterns in their six mixed gastric cases, as revealed by multiregional sampling and phylogenetic tree construction. Like in our cohort, three of their cases showed a sequential divergence pattern, with the NEC component differentiated from AC, whereas the remaining three cases showed parallel divergence, with multiple splits and NEC diverging from AC more than once (27). Whether this latter pattern of divergence may be present in some CR-MiNEN or if it is specific for gastric primaries warrants further investigation.

Since most of the private NEC branch mutations observed in this study had low VAFs, there must be a majority of NEC cells only harbouring the trunk mutations (i.e. the same mutations as found in the AC component). This means that the private mutations are most likely not drivers of the AC-to-NEC-transition in CR-MiNEN, and that the phenotypical divergence of the two components must be caused by other mechanisms than the mutations covered by the 360-cancer gene panel.

We assessed the general CNA patterns across the AC and NEC samples, in addition to investigating selected genes frequently altered in digestive NEC. We did not find any systemic difference in CNAs across both tumour components, and CNAs affecting the specific genes we investigated did not appear to account for the split between the AC and NEC components. A study on eight gastric MANEC showed both shared copy number variants, suggesting a common origin, alongside a generally higher copy number loss in the NEC than the AC component (29). However, there was a higher frequency of private mutation in the NEC compared to the AC component and, in addition, for genes frequently affected by CNAs in NEC, a higher number of CNAs was found in the NEC than in the AC components. Taken together, these findings indicate a higher mutation rate and likely a higher general genomic instability in the NEC than in the AC component. Such a pattern fits with the more aggressive phenotype of colorectal NEC than AC and may also signal an underlying cause that is related to the phenotypical shift to NEC. While we have no firm evidence for such an underlying cause, it has been previously reported that high ploidy and whole-genome duplications often occur in digestive NEC (16). In this study, the ploidy was significantly higher in the NEC components as compared to the AC components. One may, therefore, speculate if whole-genome doubling and subsequent loss of genomic stability may be an underlying contributor to the NEC phenotype.

The divergence of NEC from AC may also have several other underlying causes. It might be linked to epigenetic mechanisms such as methylation (30). Furthermore, spatial transcriptomics has revealed downregulation of immune-related pathways and upregulation of proliferation-associated pathways in the NEC compared with the AC component in three cases of MiNEN, including one case with a colon primary (31). Also, an investigation into the tumour environment of 32 gastric MANEC has revealed that the AC component had more intraepithelial infiltrating immune cells than the NEC component, suggesting that the AC component is more immunogenic (32). Further investigation into transcriptomics and the tumour microenvironment may provide a biological context for the differentiation of AC and NEC components. In future studies, multi-omics examining of both components will be essential for our understanding of the underlying biology of these tumours. For the current clinical context, a single biopsy is probably sufficient, as *BRAF* and *KRAS* mutations representing potential therapeutic targets are present in both components.

We observed parallel evolution to be a common feature. In particular, multiple *NF1* mutations were observed, restricted to the NEC components. Since these mutations had low VAF, it is not likely that they are key drivers in the divergence from AC to NEC, but it rather seems like there is a selection pressure favouring *NF1* deficiency in late NEC evolution. While there is limited information on parallel evolution in digestive NEC, few studies have analysed NEC cases with a similar mutation calling sensitivity as our present analysis and parallel evolution may have been overlooked. Notably, it has previously been reported that a strong parallel evolution in a digestive NEC case was identified by multiple low VAF *PTEN* mutations in ctDNA (33).

In two cases, study-specific histology assessment supported a diagnosis of adenocarcinoma with synaptophysin expression rather than NEC, despite previous pathological re-evaluation, illustrating the diagnostic complexity and the remaining grey zones in the field of digestive NEC and MiNEN.

### Study limitations

Although we believe this study provides critical insight into the origin and development of CR-MiNEN, the sample size was limited to ten cases. The sample size limits statistical power, and the results should be validated in a larger cohort. Some common trunk mutations show low VAF in all components, indicating that there might be cells without trunk mutations. Our data were based on a 360-cancer gene panel, and we acknowledge that whole-genome data may have resolved some of these issues and also allowed for higher resolution copy number data and subclone analyses to build phylogenetic trees. However, such analyses were precluded by the limited amounts of material and the nature of available material (FFPE).

## Conclusion

Shared mutations between the AC and NEC components in all analysed MiNEN cases strongly indicate a common clonal origin. In all cases, AC and NEC split once, with subsequent branching of the NEC component. Canonical driver mutations in colorectal tumourigenesis (*BRAF*, *KRAS*, *APC,* and *TP53*) appear to play an important role in the early development of these tumours. Private mutations generally had low VAF and cannot explain the phenotypical split. As such, the mechanisms underlying divergence and the subclonal drivers of the NEC component remain to be elucidated, but may be associated with genome doubling.

## Supplementary materials









## Declaration of interest

SM, HS, WD, HTD, AV, LWV, PP, and CK declare no conflicts of interest. AP received advisory board honoraria from Böhringer Ingelheim. SK received lecture honoraria from Novartis and Pfizer. Research support (to the institution) was provided by AstraZeneca, Pfizer, and Illumina.

## Funding

The Western Norway Regional Health Authority (grant number F-12180), the Nordic Cancer Union (grant number R280-A16006), the Norwegian Cancer Society (grant number 223271), Novartis (grant number NO1501286042), and Ipsen supported this work.

## Author contribution statement

SM conceptualized the study; curated, analysed, and interpreted the data; and wrote the original draft. HS was involved in conceptualization of the study, acquisition, curation, and interpretation of the data and wrote the original draft. WD and HTD performed the bioinformatics analysis of NGS data. AV carried out the wet laboratory procedures for tumour tissue sampling and NGS. LWV, PP, and CK acquired the data. AP carried out pathological re-evaluation. SK conceptualized the study, interpreted the data, and wrote the original draft. All authors reviewed and revised the manuscript and approved the final manuscript.

## Ethics

The NORDIC NEC study was approved by regional ethics committees in Denmark (Region Hovedstaden H-4-2012-108), Sweden (REC Uppsala Dnr 2012/285), and Norway (REK Vest 2012/940). The study was conducted in accordance with the Declaration of Helsinki, and written informed consent was obtained from all patients.

## Artificial intelligence (AI) disclosure

Grammarly was used for language editing of the manuscript. No generative AI tools were used in the preparation of the written or visual content of the manuscript.
