# Supplementary figures and images for: Origin and evolution of colorectal mixed neuroendocrine–non-neuroendocrine neoplasms (MiNEN)
Source: Endocr Relat Cancer. 2026 Jul 27;33(7):e260170. doi: 10.1530/ERC-26-0170 (PMC13428015; doi:10.1530/ERC-26-0170)

**Sampling of AC component**

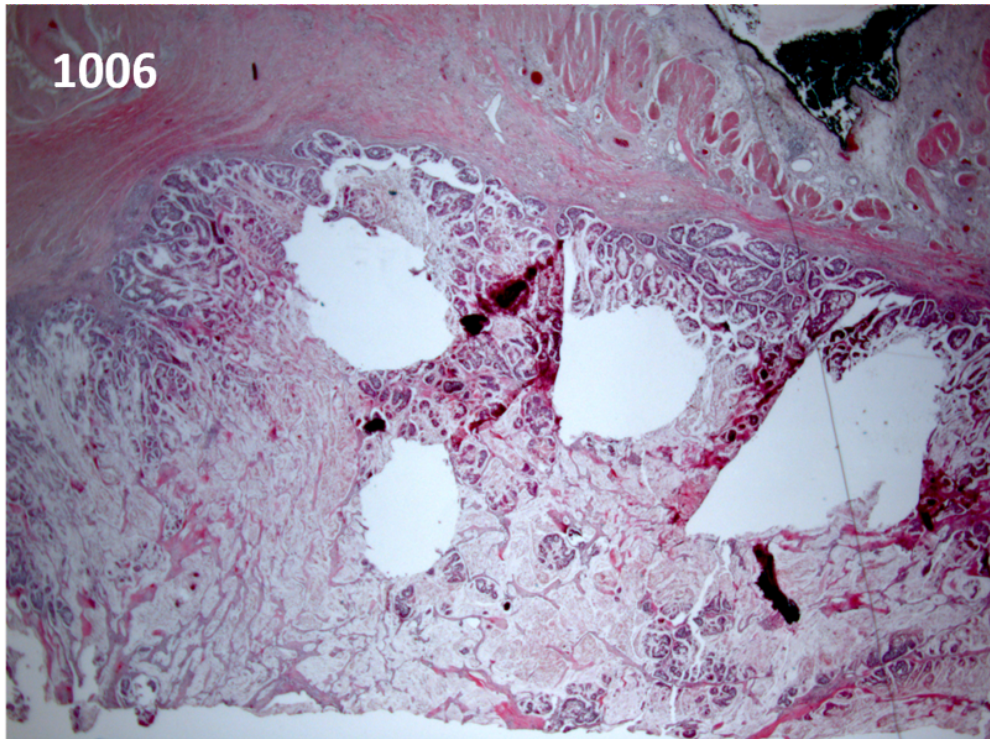

**Sampling of NEC component**

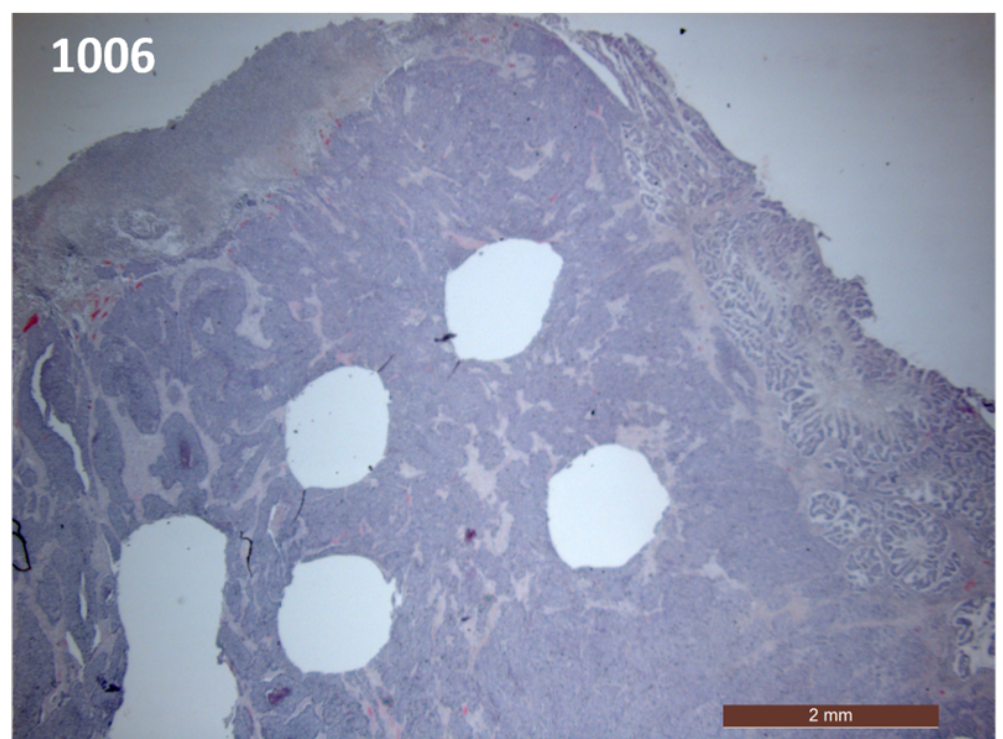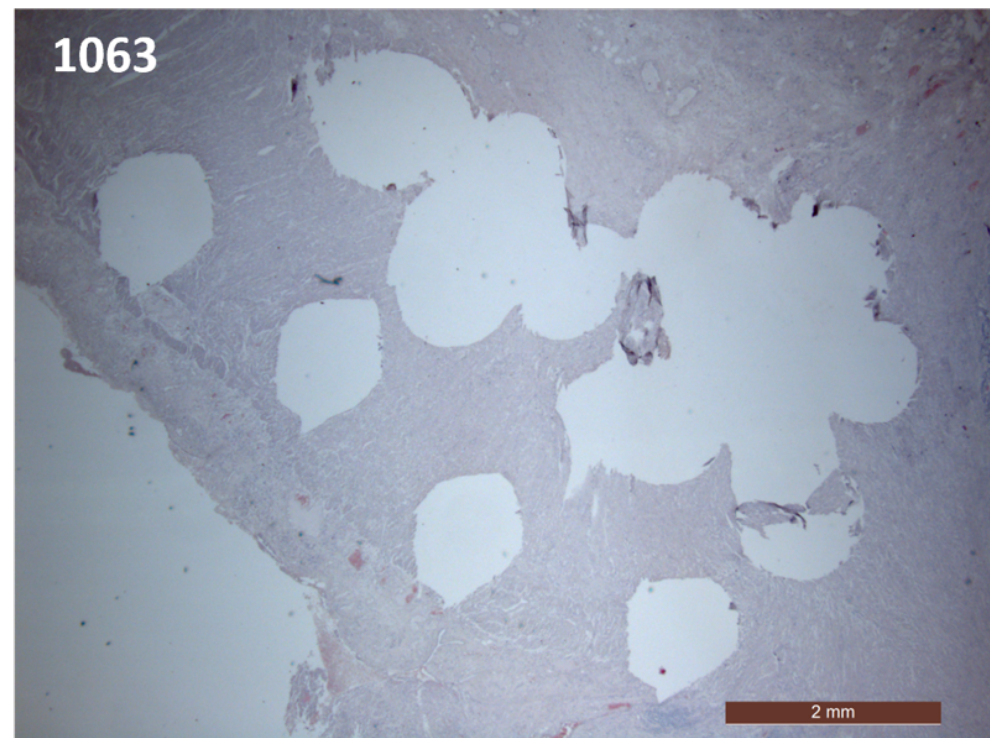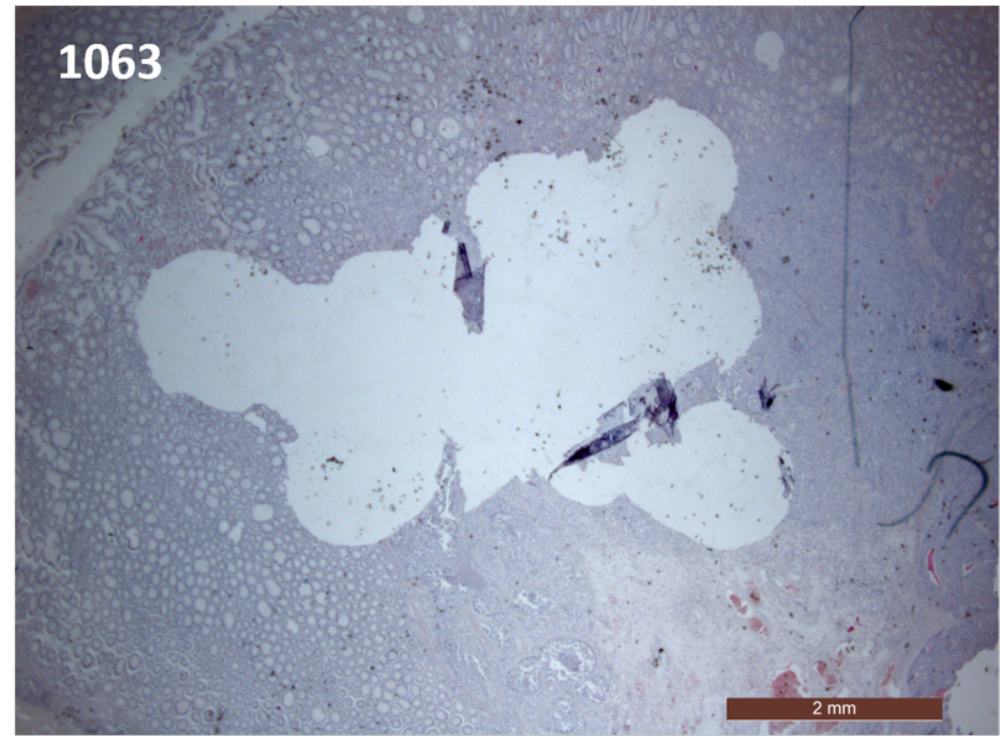

Supplement: Supplementary file 1 [file ERC-26-0170_supplementary_figure_1.pdf]

A: 1006

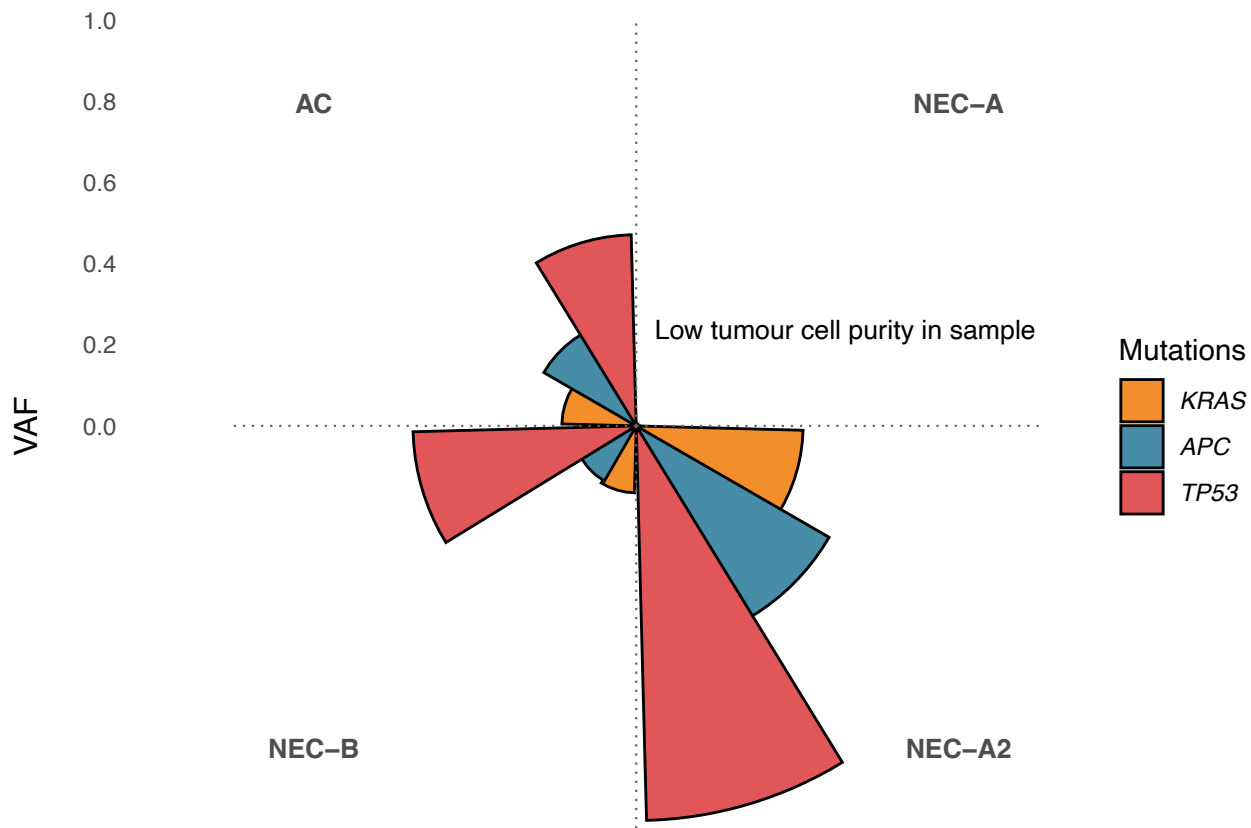

B: 1007

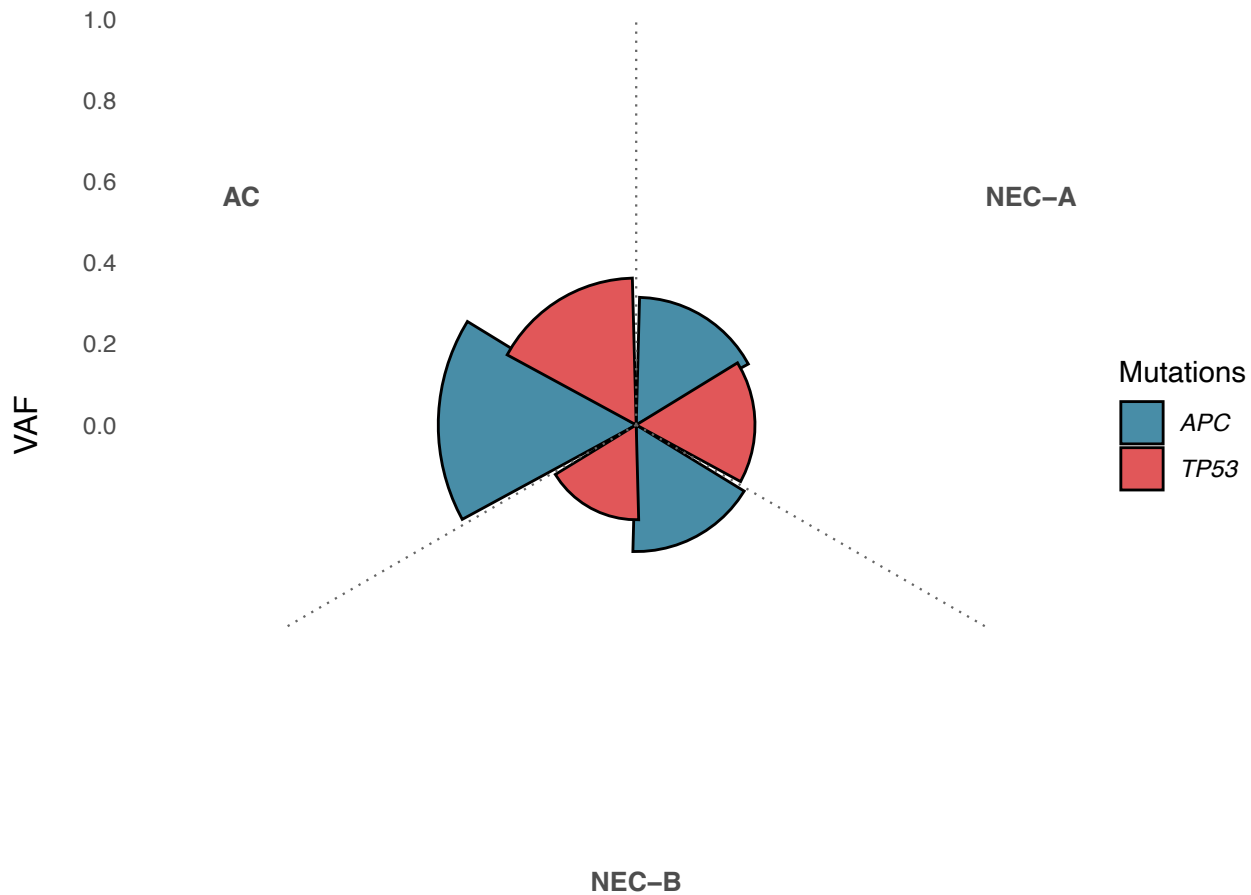

C: 1008

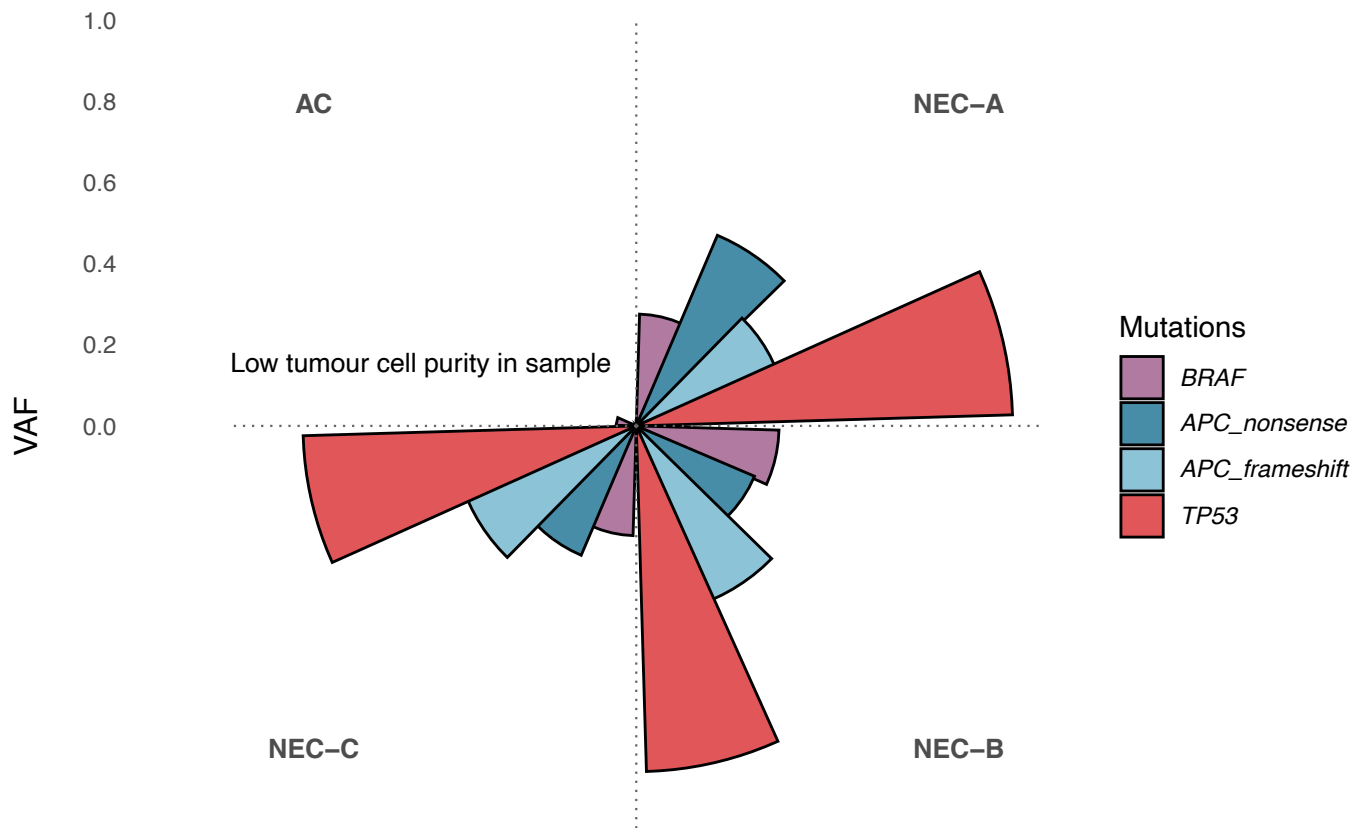

D: 1023

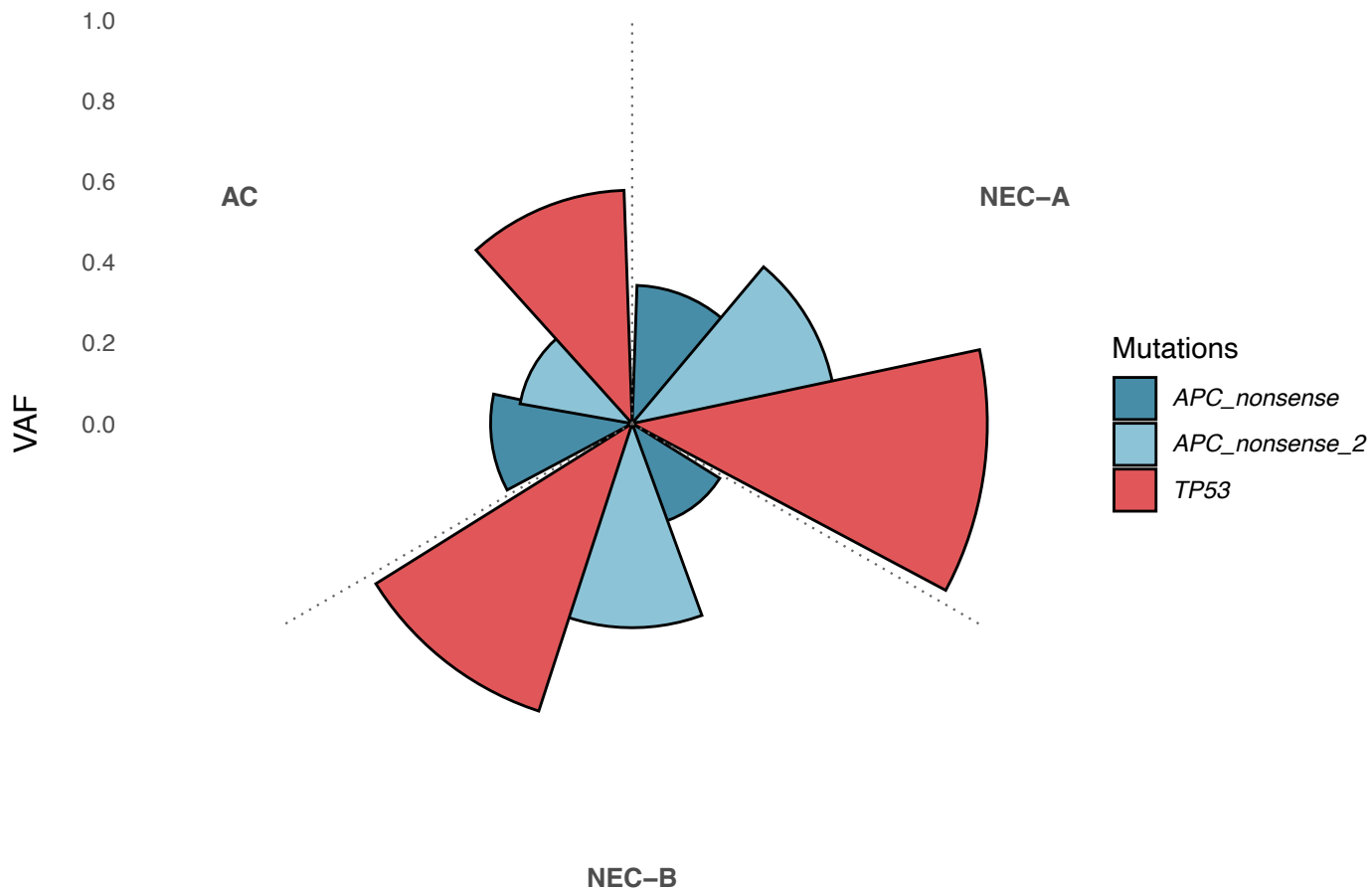

E: 1061

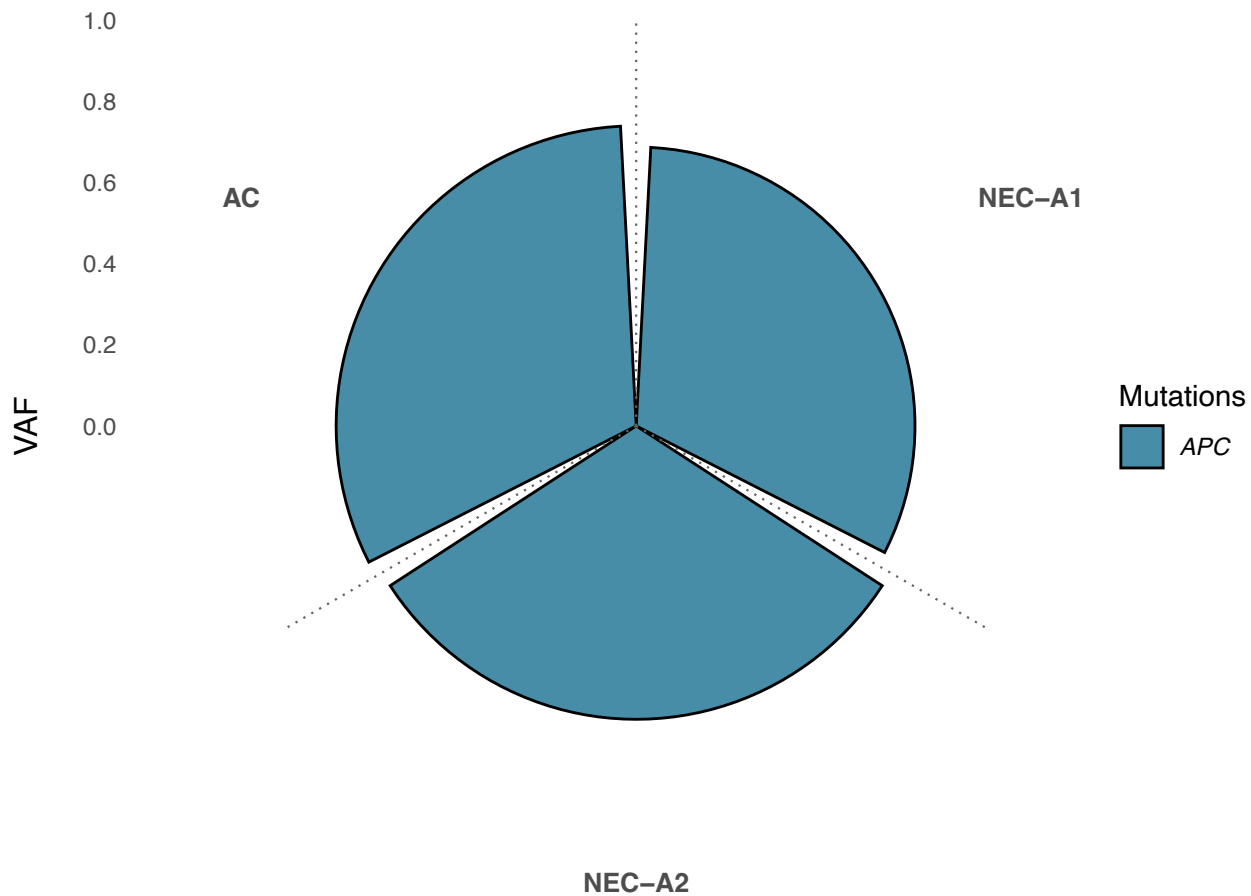

F: 1063

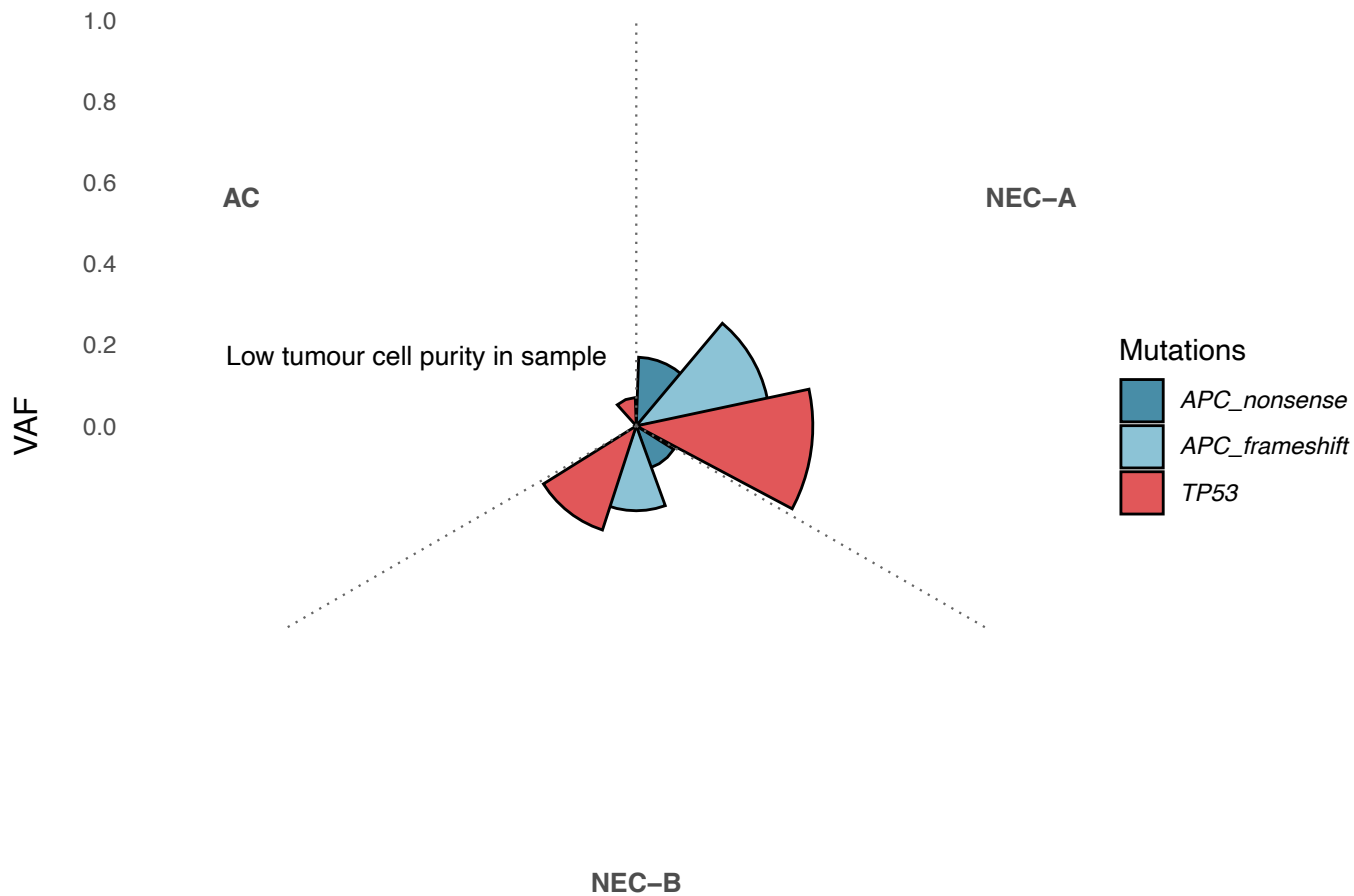

G: 8040

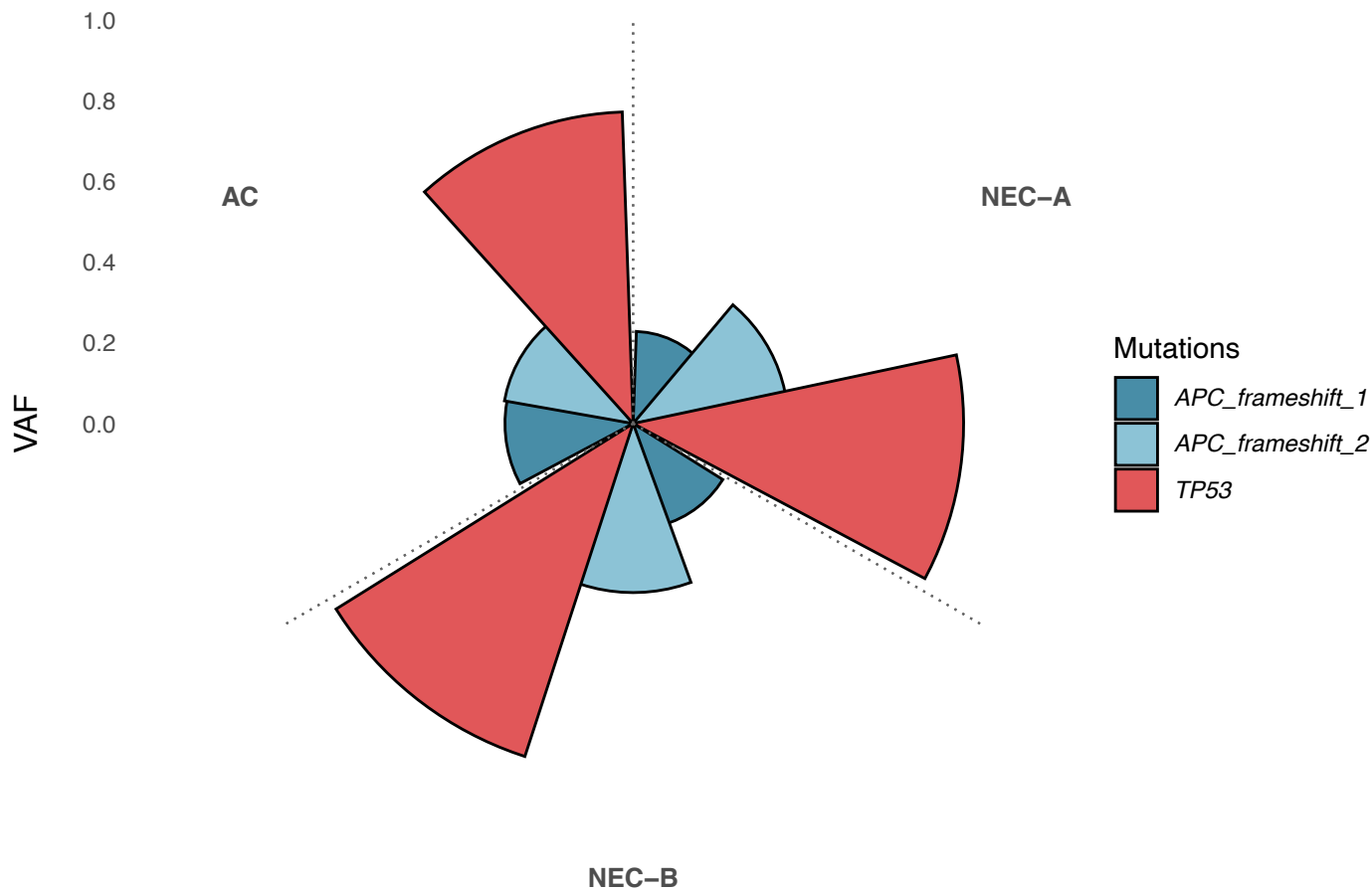

H: 8090

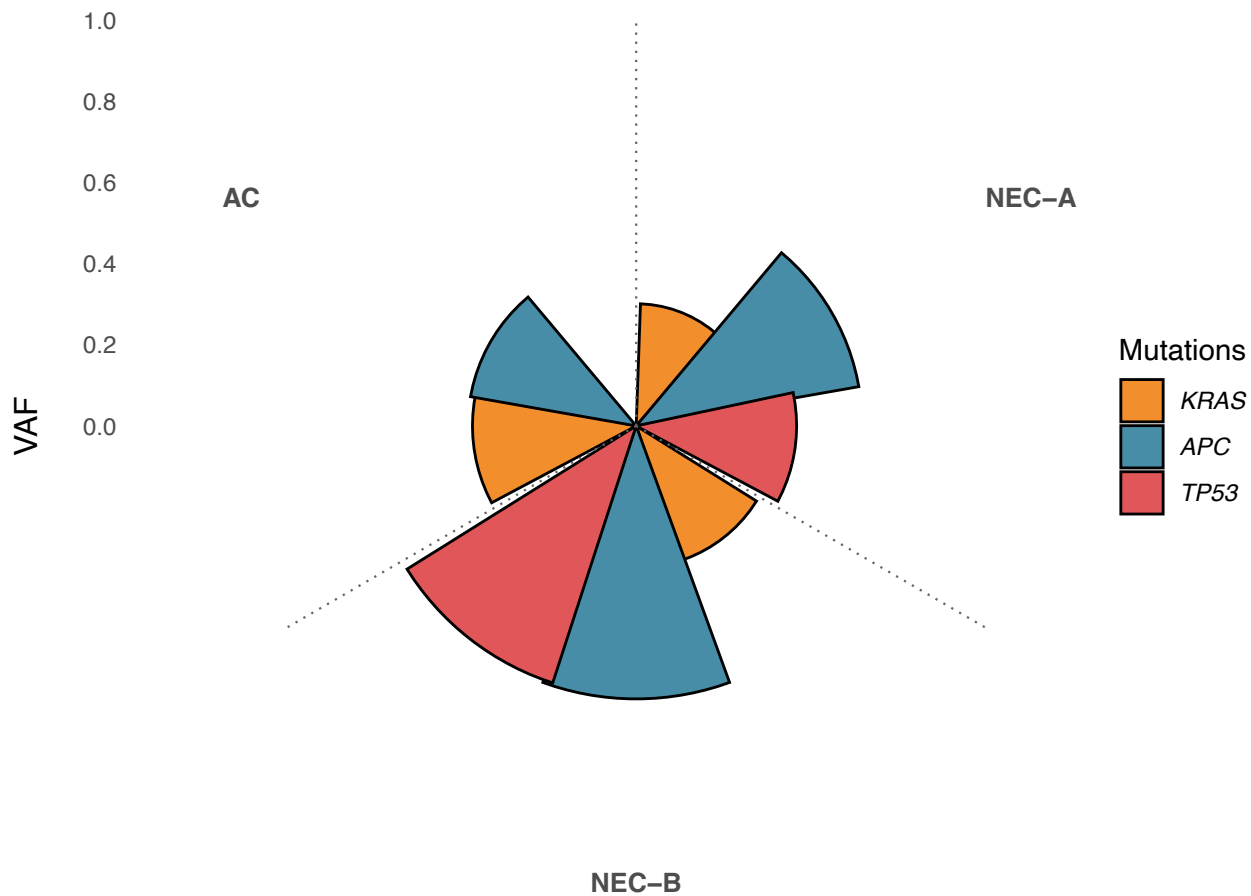

I: 11020

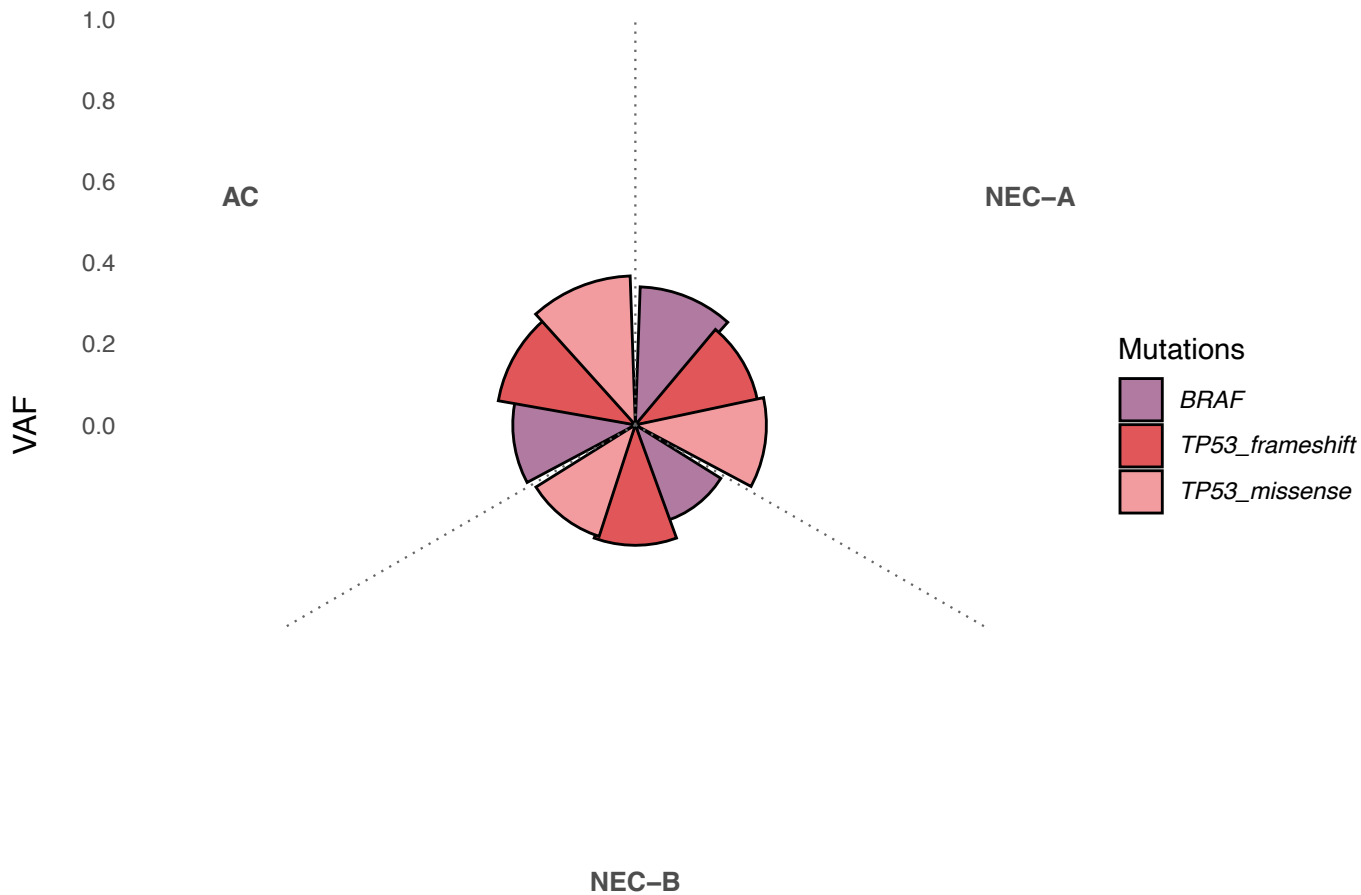

J: 11024

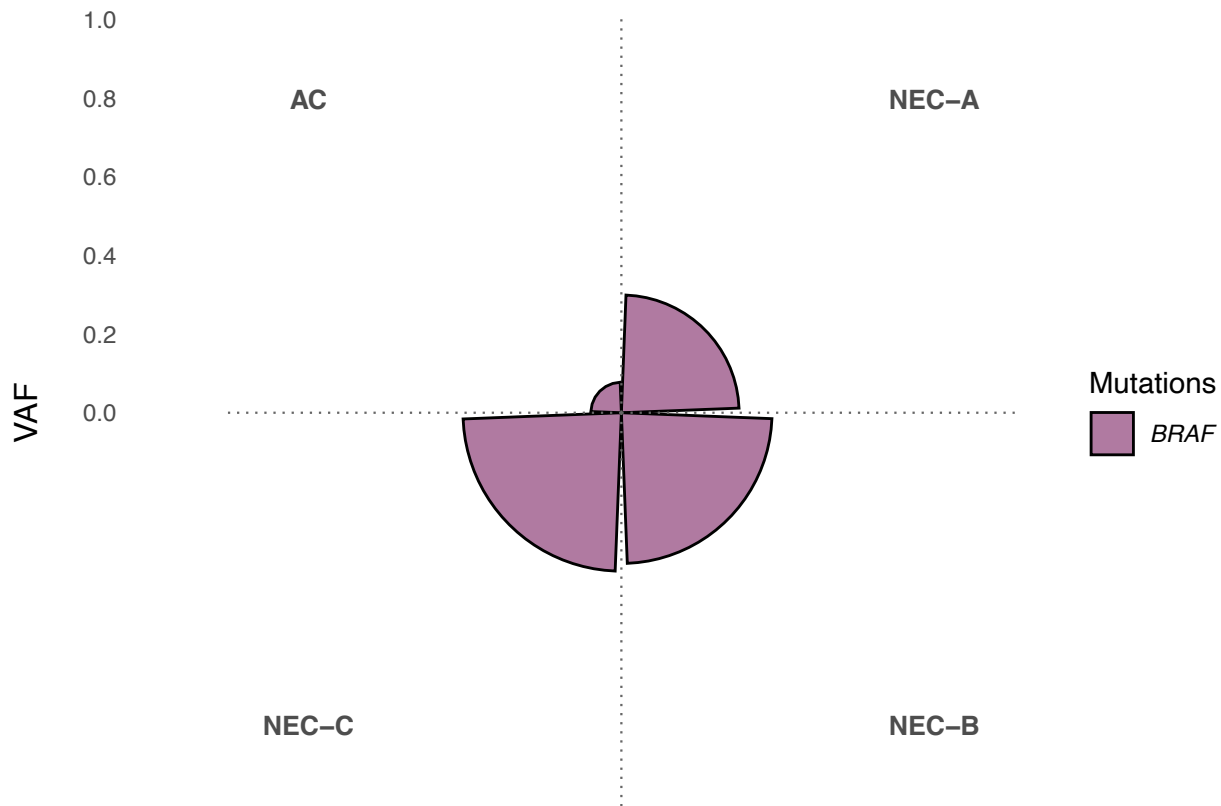

Supplement: Supplementary file 2 [file ERC-26-0170_supplementary_figure_2.pdf]

Ploidy

6

5

4

3

2

1

AC

NEC

Wilcoxon  $p = 0.047$

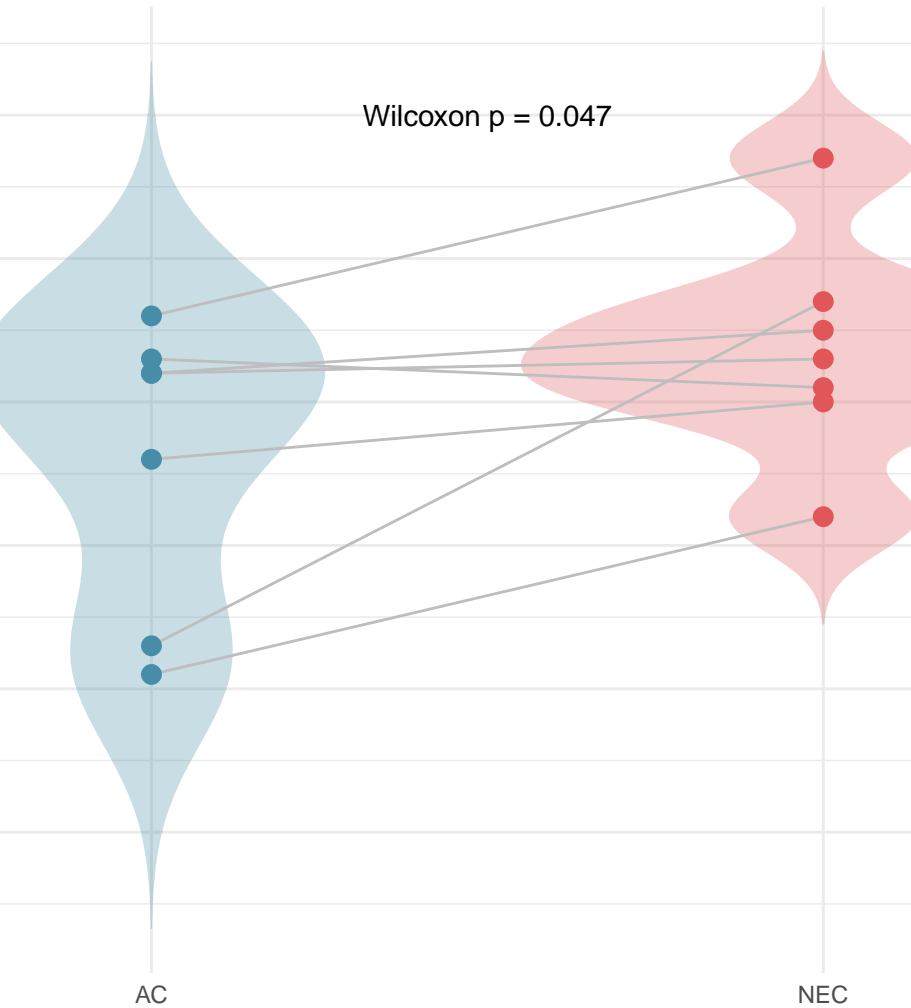

Supplement: Supplementary file 3 [file ERC-26-0170_supplementary_figure_3.pdf]
