# Supplementary material for: Origin and evolution of colorectal mixed neuroendocrine–non-neuroendocrine neoplasms (MiNEN)
Source: Endocr Relat Cancer. 2026 Jul 27;33(7):e260170. doi: 10.1530/ERC-26-0170 (PMC13428015; doi:10.1530/ERC-26-0170)
Supplement: Supplementary file 4 [file ERC-26-0170_supplementary_tables.pdf]

Supplementary Data

Sequencing

DNA isolation from formalin fixed paraffin embedded (FFPE) tumour specimens was carried out by Adaptive Focused Acoustics (AFA)-based extraction using the Covaris truXTRAC FFPE DNA kit, as previously described in detail (1). DNA from blood was used as normal reference.

Targeted massive parallel sequencing was performed. DNA from each FFPE samples was used as input in preparation of Illumina libraries applying the Kapa Hyper Prep kit (Kapa Biosystem) and the Agilent SureSelect XT-kit (Agilent Technologies, Santa Clara, CA, United States of America). Enrichment of regions of interest was performed using a custom RNA bait design (SureSelect, Agilent, UK). Regions of interest (bait targets) were an in-house cancer gene panel of 360 genes (2). Sequencing was performed on a MiSeq instrument (Illumina, San Diego, CA, United States of America). The procedures for library preparation and sequencing are described in detail in (1).

Somatic mutations were called from sequencing data in matched tumour-normal mode using the DRAGEN somatic pipeline (v3.9.5, Illumina). To minimise low-variant allele frequency (VAF) false-positive calls arising from site-specific sequencing or platform noise, a custom Systematic Noise Filtering model was constructed based on previous in-house sequencing data generated using the same 360-gene panel and the same sequencing platform. This filter was applied in combination with standard technical filtering criteria.

A mutation was retained for all samples from a given patient if it passed all filtering criteria and had a VAF ≥ 0.05 in at least one sample from that patient, while being present but not passing filters in other samples from the same patients.

Følsomhet Intern (gul)

Variants were annotated using ANNOVAR (openbioinformatics.org) against the hg38 reference genome.

MSI status was available for seven patients through our previous MSI analysis using the Promega MSI Analysis System (Version 1.2, Promega), as previously described (1). For the remaining five cases, MSI status was available from local routine diagnostics.

Copy number status, tumour ploidy and purity was assessed using the FACETS algorithm (3), as previously described for similar samples (1). For annotation of copy number alterations (CNAs) to before or after split of the AC and NEC components, copy numbers and ploidy were first forced (rounded) to integer values. Thereafter, ploidy adjusted copy numbers (copy number minus ploidy) equalling 0 in both the AC and NEC components were considered as no CNA. CNAs that were ≠ 0 and identical across an AC and a NEC sample within a patient, were defined as truncal CNAs. CNAs that were ≠ 0 in only the AC or NEC sample or was numerically different between the AC and NEC sample, were defined as a branch CNAs. Notably, a limitation with this approach is that some CNA occurring before a potential whole genome duplication may be called as branch events, while they are more likely to be trunk events and some parallel CNA in different compartments may falsely be called as truncal if preceded by a genome duplication in one of the compartments. For a subset of selected genes, copy number status (regardless of ploidy) in the AC and NEC component within a patient was assessed for direct comparison.

Assumptions for creating mock phylogenetic trees

The phylogenetic trees were constructed by assessing which mutations were shared between all samples, creating the common trunk of the tree. Thereafter, mutations present in multiple, but not all samples were used to create region-specific trunks (in the present samples, data revealed exclusively NEC-specific trunks). Lastly, private mutations present in only one sample were assessed and formed

Følsomhet Intern (gul)

the peripheral branches of the trees. In trees with logical breaks, additional strategies were applied: 1. post-hoc manual search for mutations below the original VAF, and 2. assessment of tumour cell purity when the logical break in the trees was not solved with the post-hoc mutation calling.

1006

The initial tree constructed based on the original mutation calling showed no shared trunk mutations in the NEC-A sample. The post-hoc mutation calling in the NEC-A sample identified three shared trunk mutations at low VAFs (*ERBB3* (VAF 0.023), *FBXW7* (VAF 0.003), and *NF1* (VAF 0.007)), while the other four shared trunk mutations were not identified in the sample. In addition, all private NEC-A sample mutations had low VAF. Assessment of tumour cell purity in the NEC-A sample showed a low purity below the threshold for formal calling with the FACETS algorithm. Taken together, we excluded the NEC-A sample from the tree based on low tumour cell purity.

1007:

Post-hoc mutation calling identified the presence of *CKS1B* mutation (VAF 0.017) in the NEC-A sample and *HNF1A* mutation (VAF 0.025) in the NEC-B sample, adding them to the shared trunk mutations and annotated in grey in the common trunk of the tree.

1008:

Post-hoc mutation calling identified two *NF1* mutations in the NEC-C (VAF 0.007 and 0.022) and AC (VAF 0.030 and 0.006) samples, adding them to the shared trunk mutations and annotated in grey in the common trunk of the tree. In addition, there was a logical break regarding a mutation in *KDM5A.* Post-hoc mutation calling did not detect any *KDM5A* mutations in any of the samples. It was most likely an artefact in the original mutation calling, and the mutation was excluded from the tree.

1023:

No logical breaks.

Følsomhet Intern (gul)

1061:

No logical breaks.

1063:

The original mutation calling revealed no shared trunk mutations. Post-hoc mutation calling identified a *FLT1* mutation (VAF 0.003), a *NF1* mutation (VAF 0.012), a *SFTPA1* mutation (VAF 0.026), and a *TP53* mutation (VAF 0.010) in the AC sample, adding them to the shared trunk mutations (annotated in grey font in the common trunk of the tree). The reason for low VAF in all shared trunk mutations from the AC sample is probably due to low tumour cell purity under the threshold formal calling with the FACETS algorithm.

8040:

No logical breaks.

8090:

No logical breaks.

11020:

Post-hoc mutation calling identified a *NF1* mutation in the NEC-A sample (VAF 0.060), adding the mutation to the shared trunk mutations (annotated in grey font in the common trunk of the tree).

11024:

Post-hoc mutation calling identified a *HNF1A* mutation in the NEC-A (VAF 0.032) and NEC-B (VAF 0.042) samples, adding the mutation to the shared trunk mutations (annotated in grey font in the common trunk of the tree).

Følsomhet Intern (gul)

Annotation of driver events

BRAF, *KRAS*, *APC* and *TP53* are well-known canonical driver genes in colorectal cancer oncogenesis. Mutations in these genes were annotated as likely driver mutations (**Supplementary Table 1**), based on the criteria described below:

Oncogenes *BRAF* and *KRAS*: Canonical oncogenic mutations in recurrent hotspots: *BRAF* V600E, *KRAS* codons 12 and 13, or recurrent somatic mutations previously confirmed at this locus in the COSMIC database with a mutation count of ≥ 10 for *BRAF* and ≥ 10 for *KRAS*.

Tumour suppressor genes *APC* and *TP53*: Truncating (nonsense and frameshift) mutations and in-frame insertions and deletions were considered driver events, along with somatic mutations previously confirmed at this locus in the COSMIC database with a mutation count of ≥ 10 for APC. Additionally, all missense mutations in *TP53* were considered driver events. We had no somatic *TP53* missense mutations in codons 72 (which is known polymorphic germline site). Splice-site mutations were considered a driver event when detected +/- 3 base pairs from splice site (two observed cases for *TP53*). Silent mutations (synonymous) were not considered driver events.

Supplementary Table 1. Annotation of drivers among mutations in the key genes *BRAF*, *KRAS*, *APC* and *TP53*, in the present samples set.

Gene	Patient	Mutation	Driver	Comment	Location in tree
BRAF	1008	V600E	yes	Known hotspot	Common trunk
	11020	V600E	yes	Known hotspot	Common trunk
	11024	V600E	yes	Known hotspot	Common trunk
KRAS	1006	G13D	yes	Known hotspot	Common trunk
	8090	G12D	yes	Known hotspot	Common trunk

Følsomhet Intern (gul)

APC	1006	Y997X	yes	Nonsense	Common trunk
	1007	S1356X	yes	Nonsense	Common trunk
	1008	E763X	yes	Nonsense	NEC trunk
	1008	T1556Nfs*3	yes	Frameshift	Common trunk
	1023	S1222X	yes	Nonsense	Common trunk
	1023	Q1291X	yes	Nonsense	Common trunk
	1061	E1155X	yes	Nonsense	Common trunk
	1061	D1394Y	no	Missense	Common trunk
	1063	C451X	yes	Nonsense	NEC trunk
	1063	W1577Dfs*73	yes	Frameshift	NEC trunk
	8040	R1158Tfs*5	yes	Frameshift	Common trunk
	8040	T1487Ifs*17	yes	Frameshift	Common trunk
	8090	C1387*	yes	Frameshift	Common trunk
TP53	1006	E286K	yes	Missense	Common trunk
	1007	G245S	yes	Missense	Common trunk
	1008	R273H	yes	Missense	NEC trunk
	1023	C277F	yes	Missense	Common trunk
	1063	R196X	yes	Nonsense	Common trunk
	8040	R175H	yes	Missense	Common trunk
	8090	R273P	yes	Missense	NEC trunk
	11020	H178Tfs*69	yes	Frameshift	Common trunk
	11020	R273C	yes	Missense	Common trunk

Supplementary Table 2: Copy number alterations (CNAs) patterns across the gene panel. Ploidy-adjusted CNAs occurring before (truncal CNAs) and after (branch CNAs) the split of the adenocarcinoma (AC) and neuroendocrine carcinoma (NEC) component in 10 cases with colorectal mixed neuroendocrine non-neuroendocrine neoplasm.

	**No CNAs**	**Truncal CNAs**	**Branch CNAs**	**Distribution of branch AC and NEC CNAs**
**1006**				
AC and NEC-A*	0	0	356/356 (100%)	340 and 152
AC and NEC-A2	0	77/356 (22%)	279/356 (78%)	263 and 235
AC and NEC-B	0	160/356 (45%)	196/356 (55%)	180 and 131
Mean**	0%	33%	67%	
**1007**				
AC and NEC-A	79/355 (22%)	141/355 (40%)	135/355 (38%)	97 and 84
AC and NEC-B	39/355 (11%)	192/355 (54%)	124/355 (35%)	46 and 124
Mean	17%	47%	36%	
**1008**				
AC* and NEC-A	103/355 (29%)	18/355 (5%)	234/355 (66%)	126 and 206

Følsomhet Intern (gul)

AC* NEC-B	56/355 (16%)	10/355 (3%)	289/355 (81%)	134 and 225
AC* and NEC-C	79/355 (22%)	41/355 (12%)	235/355 (66%)	103 and 202
Mean	22%	7%	71%	
**1023**				
AC and NEC-A	43/356 (12%)	96/356 (27%)	217/356 (61%)	217 and 84
AC and NEC-B	29/356 (8%)	168/356 (47%)	159/356 (45%)	145 and 100
Mean	10%	37%	53%	
**1061**				
AC and NEC-A1	19/356 (5%)	130/356 (37%)	207/356 (58%)	157 and 193
AC and NEC-A2	50/356 (14%)	180/356 (51%)	126/356 (35%)	107 and 102
Mean	9%	44%	47%	
**1063**				
AC* and NEC-A	11/356 (3%)	9/356 (3%)	336/356 (94%)	125 and 315
AC* and NEC-B	0	0	356/356 (100%)	134 and 356
Mean	2%	1%	97%	
**8040**				
AC and NEC-A	0	119/355 (34%)	236/355 (66%)	216 and 159
AC and NEC-B	11/355 (3%)	80/355 (23%)	264/355 (74%)	255 and 203
Mean	2%	28%	70%	
**8090**				
AC and NEC-A	19/355 (5%)	9/355 (3%)	327/355 (92%)	308 and 246
AC and NEC-B	0	64/355 (18%)	291/355 (82%)	253 and 282
	3%	10%	87%	
**11020**				
AC and NEC-A	49/356 (14%)	20/356 (6%)	287/356 (80%)	104 and 238
AC and NEC-B	190/356 (53%)	103/356 (29%)	63/356 (18%)	21 and 54
Mean	34%	17%	49%	
**11024**				
AC and NEC-A	97/356 (27%)	90/356 (25%)	169/356 (47%)	68 and 169
AC and NEC-B	68/356 (19%)	51/356 (14%)	237/356 (67%)	107 and 186
AC and NEC-C	90/356 (25%)	98/356 (28%)	168/356 (47%)	60 and 147
Mean	24%	22%	54%	

* Tumour cell purity under threshold for FACETS

** NEC-A excluded from the calculation

Supplementary figure legends

Supplementary Figure 1. Histology. Haematoxylin and eosin slides from two cases (1006 and 1063) illustrating sampling from the adenocarcinoma (AC) neuroendocrine carcinoma (NEC) component of the tumour.

Supplementary Figure 2. Colorectal driver mutations. Plots illustrating the distribution of *BRAF* (purple), *KRAS* (orange), *APC* (blue), and *TP53* (red) mutations in 10 cases with colorectal mixed neuroendocrine-none-neuroendocrine neoplasm. Each segment illustrates a specific mutation with the radial extent reflecting the variant allele frequency of the mutation.

REFERENCES

1. Venizelos A, Elvebakken H, Perren A, et al. The molecular characteristics of high-grade gastroenteropancreatic neuroendocrine neoplasms. Endocr Relat Cancer. 2021;29(1):1–14.

2. Yates LR, Gerstung M, Knappskog S, et al. Subclonal diversification of primary breast cancer revealed by multiregion sequencing. Nat Med. 2015;21(7):751–9.

3. Shen R, Seshan VE. FACETS: allele-specific copy number and clonal heterogeneity analysis tool for high-throughput DNA sequencing. Nucleic Acids Res. 2016;44(16):e131.

Følsomhet Intern (gul)
